# Trends and disparities in disease burden of age-related macular degeneration from 1990 to 2019: Results from the global burden of disease study 2019

**DOI:** 10.3389/fpubh.2023.1138428

**Published:** 2023-04-17

**Authors:** Bo Jiang, Chun Jiang, Jianqing Li, Peirong Lu

**Affiliations:** Department of Ophthalmology, The First Affiliated Hospital of Soochow University, Suzhou, Jiangsu, China

**Keywords:** age-related macular degeneration, trends, disparities, global burden of disease study, prevalence

## Abstract

**Objectives:**

This study aims to estimate the trends and disparities in the worldwide burden for health of AMD, overall and by age, sex, socio-demographic index (SDI), region, and nation using prevalence and years lived with disability (YLDs) from Global Burden of Disease (GBD) study 2019.

**Methods:**

This retrospective study presents the prevalent AMD cases and YLDs from 1990–2019, as well as the age-standardized prevalence rate (ASPR) and age-standardized YLD rate (ASYR) of AMD. To measure changes over time, estimated annual percentage changes (EAPCs) of the age-standardized rates (ASRs) were analyzed globally, then studied further by sex, SDI, region, and nation. We included data from the 2019 Global Burden of Disease (GBD) database to examine AMD prevalence and YLDs from 1990–2019 in 204 countries and territories, as well as demographic information such as age, sex, SDI, region, and nation.

**Results:**

Globally, the number of prevalent AMD cases increased from 3,581,329.17 (95% uncertainty interval [UI], 3,025,619.4–4,188,835.7) in 1990 to 7,792,530 (95% UI, 6,526,081.5–9,159,394.9) in 2019, and the number of YLDs increased from 296,771.93 (95% uncertainty interval [UI], 205,462.8–418,699.82) in 1990 to 564,055.1 (95% UI, 392,930.7–789,194.64) in 2019. The ASPR of AMD had a decreased trend with an EAPC of −0.15 (95% confidence interval [CI], −0.2 to −0.11) from 1990 to 2019, and the ASYR of AMD showed a decreased trend with an EAPC of −0.71 (95% confidence interval [CI], −0.78 to −0.65) during this period. The prevalence and YLDs of AMD in adults over 50 years of age showed a significant increase. The prevalence and YLDs of AMD were significantly higher in females than males, overall. The ASPRs and ASYRs in low SDI regions was greater than in high SDI regions from 1990 to 2019. In addition, increases in prevalence and YLDs differed by regions and nations, as well as level of socio-economic development.

**Conclusion:**

The number of prevalent cases and YLDs due to AMD increased over 30 years and were directly linked to age, sex, socio-economic status, and geographic location. These findings can not only guide public health work but also provide an epidemiological basis for global strategy formulation regarding this global health challenge.

## Introduction

Globally, age-related macular degeneration (AMD) is a leading cause of irreversible vision loss in many patients with posterior segment eye diseases (1, 2). AMD has imposed a heavy burden on these individuals and society: poor vision due to AMD can cause negative consequences, such as increased risk of falls, depression, lack of meaningful activities in daily life, and requirement of long-term care (3–5). More importantly, the overall aging of the population means that the cases of AMD have risen exponentially in recent years. The number of patients affected by AMD is expected to increase globally, from around 200 million in 2020 to nearly 300 million in 2040 (6, 7). Vision complications from AMD can significantly affect the quality of life in older adults and contribute to already-challenging health problems and comorbidities (8, 9). Understanding the AMD disease burden over time (and any influencing factors) may aid in formulating targeted public policies and reducing the public disease burden.

Many population-based AMD studies have been conducted worldwide. A cohort study reported that the incidence rate of early AMD was highest among White people (5.3%), medium among Chinese (4.5%) and Spanish people (3.3%), and the lowest among Black people (1.6%). The incidence rate of late AMD was similar; 4.1, 2.2, 0.8, and 0.4%, respectively (10). One meta-analysis of studies with European populations indicated that the prevalence of early or late AMD was lower in the group of 50–55 year old participants (0.08%) than in the group of 90+ year old participants (20.1%) (11). Another meta-analysis showed that in Asian participants aged 40–79 years, early AMD prevalence was 6.8% and late AMD prevalence was 0.56%; the corresponding prevalence in the White participants of the same age range were 8.8 and 0.59%, respectively (12). Although there have been several studies on the disease burden of AMD, few studies described the prevalence of the condition globally, across geographical regions, over time, and any overall influencing factors (13–15).

The 2019 Global Burden of Disease Study (GBD 2019) is the latest published data from 204 countries and territories, and it includes the most recent estimates of disease burden and data of influencing factors for researchers to study. The GBD database (GBDx) is essential for understanding the global burden of many diseases and Global Burden of Disease Study in 2019 (GBD 2019) is the most updated and comprehensive systematic study on the prevalence and years lived with disability (YLDs) of AMD. The purpose of this study is to understand the global, regional, and national prevalence and YLDs of AMD and influences on this prevalence and YLDs. In this study, we investigated the changes in the global prevalence and YLDs of AMD from 1990 to 2019 and assessed the global burden of AMD by age, sex, socio-demographic index (SDI), region, and nation. Our findings can not only guide public health work, but also provide an epidemiological basis for global strategy formulation regarding AMD burden.

## Methods

### Data

Our global burden data on AMD are from the GBD 2019 which is freely available; therefore, no ethical approval or informed consent was required. These data include the estimated number of prevalent AMD cases and YLDs each year, age-standardized prevalence rate (ASPR), age-standardized YLD rate (ASYR), and change over time by age, sex, SDI, region, and nation from 1990 to 2019. Results are presented as number of prevalent cases, ASPR, ASYR and the estimated annual percentage change (EAPC), using 95% uncertainty intervals (UI) and 95% confidence intervals (CI).

### Factor calculations

The Disease Modeling-Meta Regression (DisMod-MR) version 2.1 was used to model the epidemiological outcomes of AMD, a Bayesian meta-regression framework widely used for GBD epidemiological modeling. The general methods and disease burden estimation methods of GBD 2019 have been previously reported (16). The prevalent cases, YLDs, prevalence and YLD rate of AMD were reported from the GBDx and stratified by age, sex, SDI, region, and nation.

SDI assignment is based on the country’s *per capita* income distribution, average years of education and the fertility rate of women younger than 25 years old. The GBDx divided the countries and territories into five SDI levels (high, high-middle, middle, low-middle, and low). In this study, the SDI ranged from 0 to 1 (low to high) and was used to describe the development level of a country or geographical region. The GBDx also grouped the countries and territories into 21 regions, the details of which have been previously reported (16–18). The disability-adjusted life-years (DALYs) are the sum of YLDs and years of life lost (YLLs) due to premature death. As for AMD, DALYs generally mean YLDs. Trends of AMD were quantified using ASPR, ASYR and EAPC. When considering variations in the age structure, it was necessary to standardize the ASR calculation (18). The ASR was obtained by the formula:


$$ASR\;\left(\mathrm{per}\;100,000\;\mathrm{population}\right)=\frac{\sum \frac{A}{i=1}\;aiwi}{\sum \frac{A}{i=1}\;wi}\times 100,000$$

The EAPC was used to explain the ASR change trend over time. A linear relationship between ASR and time can be seen in its natural logarithm. The natural logarithm rate that a regression line fit is given by the equation y = α + βx + ɛ, where y = ln (ASR), and x = calendar year. The EAPC was calculated as 100 × (exp(β) − 1). The linear regression model was used to obtain the EAPC’s 95% CI (17). All data visualization was completed using the R program (version 4.2.2) and GraphPad Prism 9.0 statistics software.

## Results

### Global AMD burden

**Figure 1:**
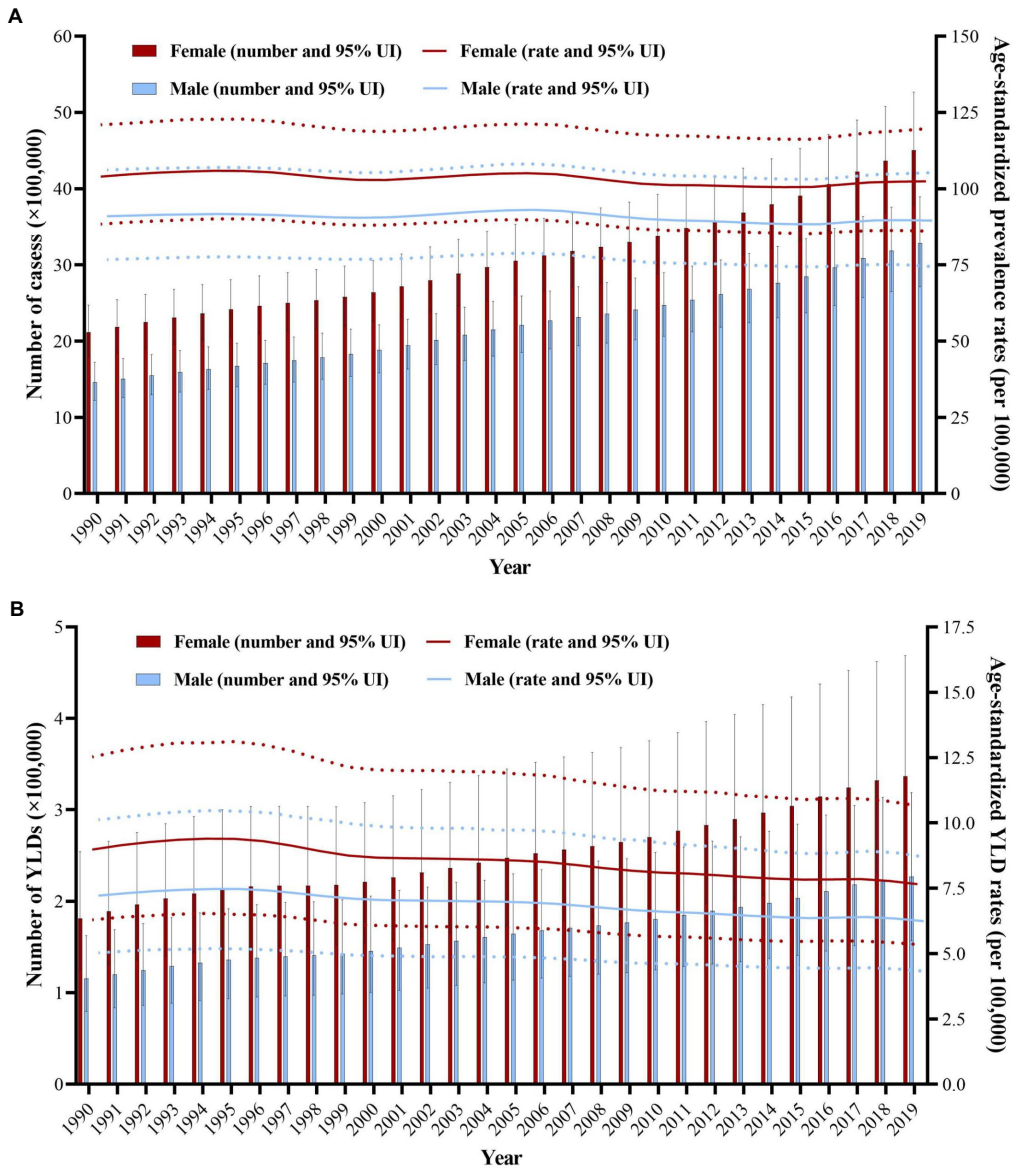
Changes in global burden of the prevalence and YLDs of AMD from 1990 to 2019. **(A)** The number of cases and age-standardized prevalence rates of AMD from 1990 to 2019. **(B)** The number of YLDs and age-standardized YLD rates of AMD from 1990 to 2019. Red and blue dashed line indicates the upper and lower limits of the 95% uncertainty intervals (95% UIs) for females and males, respectively. AMD, age-related macular degeneration; UI, uncertainty interval; YLDs, years lived with disability.

The number of prevalent AMD cases increased from 3,581,329.17 (95% uncertainty interval [UI], 3,025,619.4–4,188,835.7) in 1990 to 7,792,530 (95% UI, 6,526,081.5–9,159,394.9) in 2019, and the number of YLDs increased from 296,771.93 (95% uncertainty interval [UI], 205,462.8–418,699.82) in 1990 to 564,055.1 (95% UI, 392,930.7–789,194.64) in 2019 (Tables 1, 2). However, the ASPR of AMD dropped from 98.8 (95% UI, 83.7–114.6) per 100,000 people in 1990 to 96.8 (95% UI, 81.3–113.2) per 100,000 people in 2019, with an EAPC of −0.15 (95% CI, −0.2 to −0.11), and the ASYR of AMD dropped from 8.29 (95% UI, 5.8–11.58) per 100,000 people in 1990 to 7.05 (95% UI, 4.92–9.84) per 100,000 people in 2019, with an EAPC of −0.71 (95% CI, −0.78 to −0.65). Despite the decrease in overall ASPR and ASYR, the number of cases and YLDs due to AMD revealed increasing trends that changed between 1990 and 2019 (Figures 1A,B).

**Table 1:** Estimated number and age-standardized rate (per 100,000 persons) of prevalence and temporal trends for AMD from 1990 to 2019 by sex, SDI, and GBD regions.

Characteristics	1990		2019		1990–2019
	Number (95% UI)	ASR (95% UI)	Number (95% UI)	ASR (95% UI)	EAPC (95% CI)
Global	3,581,329.17 (3,025,619.44–4,188,835.66)	98.78 (83.72–114.63)	7,792,530.04 (6,526,081.5–9,159,394.94)	96.76 (81.32–113.2)	−0.15 (−0.2—0.11)
Sex					
Female	2,118,523.4 (1,791,457–2,473,214.1)	103.97 (88.4–121)	4,506,554.63 (3,789,113.1–5,266,137.2)	102.42 (86.1–119.7)	−0.15 (−0.2—0.11)
Male	1,462,805.76 (1,221,218.7–1,724,282.3)	90.94 (76.8–106.1)	3,285,975.41 (2,715,084.1–3,893,320)	89.55 (74.5–105.3)	−0.1 (−0.15—0.06)
SDI region					
High SDI	5,89,371.09 (4,95,590.14–6,87,094.36)	55.48 (47.03–64.67)	1,016,422.12 (8,57,435.16–1,185,659.08)	48.37 (40.74–56.29)	−0.53 (−0.59—0.46)
High-middle SDI	9,74,824.6 (8,22,768.73–1,140,725.42)	99.43 (84.57–115.34)	2,170,539.67 (1,834,349.22–2,543,933.37)	106.02 (89.95–123.71)	0.21 (0.15–0.28)
Middle SDI	1,033,914.12 (8,53,461.05–1,226,652.56)	115.34 (96.54–135.56)	2,612,130.53 (2,156,473.44–3,116,298.3)	111.12 (92.18–131.19)	−0.25 (−0.32—0.18)
Low-middle SDI	6,94,714.54 (5,70,668.71–8,22,902.11)	132.95 (111.23–156.27)	1,362,695.17 (1,122,775.79–1,621,775.89)	107.55 (89.44–127.05)	−0.9 (−0.97—0.83)
Low SDI	2,87,161.52 (2,39,354.86–3,39,032.18)	141.57 (119.62–165.63)	6,28,003.99 (5,21,118.82–7,47,865.96)	139.46 (116.25–164.76)	−0.14 (−0.19—0.09)
South–East Asia, East Asia, and Oceania					
East Asia	8,99,060.25 (7,31,153.2–1,079,383.03)	118.47 (98.5–140.87)	2,633,284.4 (2,160,679.12–3,154,893.43)	128.83 (106.73–153.26)	0.21 (0.06–0.36)
Southeast Asia	2,21,497.53 (1,83,008.36–2,64,015.65)	100.04 (83.5–117.72)	4,69,432.79 (3,90,412.44–5,57,154.75)	84.69 (70.55–99.84)	−0.75 (−0.82—0.68)
Oceania	855.47 (675.02–1,054.89)	35.46 (28.73–42.89)	1,857.22 (1,453.94–2,300.81)	32.74 (26.39–39.96)	−0.18 (−0.21—0.16)
Sub-Saharan Africa					
Eastern Sub-Saharan Africa	86,940.82 (72,271.67–1,02,765.09)	136.98 (114.83–161.38)	1,66,809.58 (1,36,759.2–1,99,384.13)	121.92 (101.38–144.97)	−0.22 (−0.29—0.16)
Central Sub-Saharan Africa	4,916.47 (3,827.5–6,163.78)	29.31 (23.31–35.76)	12,022.37 (9,520.59–14,777.57)	30.94 (24.56–37.73)	0.26 (0.23–0.29)
Southern Sub-Saharan Africa	10,093.32 (8,289.38–12,031.54)	42.05 (34.77–49.95)	22,310.37 (18,139.13–26,708.64)	45.6 (37.46–54.38)	0.37 (0.29–0.45)
Western Sub-Saharan Africa	1,66,710.92 (1,37,627.01–1,98,928.82)	210 (174.12–247.64)	3,59,408.81 (2,92,801.3–4,36,020.69)	219.31 (179.65–262.11)	0.03 (−0.06—0.13)
South Asia					
South Asia	7,46,805.46 (6,10,989.39–8,94,878.54)	154.5 (128.24–182.79)	1,499,084.21 (1,227,077.74–1,798,350.9)	114.72 (94.59–136.68)	−1.41 (−1.55—1.27)
Latin America and Caribbean					
Caribbean	6,064.03 (4,840.04–7,421.13)	24.31 (19.47–29.6)	11,526.89 (9,272.32–14,073.93)	22.29 (17.9–27.25)	−0.24 (−0.27—0.21)
Central Latin America	44,019.28 (35,831.2–52,938.98)	60.08 (49.01–71.73)	1,20,607.08 (98,249.09–1,44,367.12)	53.36 (43.57–63.61)	−0.32 (−0.35—0.29)
Tropical Latin America	68,381.04 (56,261.86–82,040.25)	83.77 (69.15–99.38)	1,93,602.3 (1,61,055.55–2,30,808.98)	82.06 (68.01–97.85)	0.18 (0.01–0.34)
Andean Latin America	21,698.24 (17,766.47–26,067.51)	116.27 (95.42–139.18)	61,461.71 (50,861.49–73,157.27)	113.58 (94.1–135.01)	−0.09 (−0.2—0.02)
North Africa and Middle East					
North Africa and Middle East	184.2 (153.9–217.8)	184.2 (153.9–217.8)	6,41,221.43 (5,28,275–7,62,563.1)	168.45 (139.4–199.2)	−0.21 (−0.27—0.16)
Central Europe, Eastern Europe, and Central Asia					
Central Asia	32,302.84 (26,043.92–39,734.46)	75.27 (60.84–92.16)	46,946.53 (37,395.69–57,823.12)	73.9 (59.71–90.29)	−0.12 (−0.16—0.08)
Eastern Europe	74,578.23 (60,736.8–89,143.14)	27.83 (22.8–33.02)	92,659.99 (76,042.2–1,09,661.05)	25.9 (21.29–30.65)	−0.33 (−0.37—0.29)
Central Europe	95,775.52 (78,777.33–1,13,942.37)	66.62 (55.04–79)	1,43,534.34 (1,16,963.63–1,72,033.35)	63.26 (51.59–75.97)	−0.21 (−0.24—0.18)
High–income regions					
Southern Latin America	15,307.08 (12,211.59–18,588.54)	35.63 (28.85–43.12)	26,823.92 (21,547.5–32,523.97)	31.2 (25.06–37.81)	−0.42 (−0.44—0.4)
Western Europe	6,48,357.13 (5,46,249.76–7,59,665.06)	107.85 (91.59–125.39)	9,76,388.69 (8,25,781.49–1,138,807.18)	93.13 (78.71–108.25)	−0.54 (−0.56—0.52)
North America					
Australasia	9,754.96 (8,011.3–11,565.82)	42.92 (35.44–50.82)	20,593.54 (17,012.15–24,503.01)	37.96 (31.26–45.24)	−0.4 (−0.47—0.32)
Asia Pacific	40,983.6 (33,396.48–49,283.4)	22.22 (18.27–26.57)	1,05,854.63 (87,551.31–1,25,058.63)	19.69 (16.3–23.36)	−0.43 (−0.54—0.32)

AMD, age-related macular degeneration; SDI, socio-demographic index; UI, uncertainty interval; CI, confidence interval; ASR, age-standardized rate; EAPC, estimated annual percentage change; GBD, global burden of disease.

**Table 2:** Estimated number and age-standardized rate (per 100,000 persons) of YLDs and temporal trends for AMD from 1990 to 2019 by sex, SDI, and GBD regions.

Characteristics	1990		2019		1990–2019
	Number (95% UI)	ASR (95% UI)	Number (95% UI)	ASR (95% UI)	EAPC (95% CI)
Global	2,96,771.93 (2,05,462.8–4,18,699.82)	8.29 (5.8–11.58)	5,64,055.1 (3,92,930.7–7,89,194.64)	7.05 (4.92–9.84)	−0.71 (−0.78—0.65)
Sex					
Female	1,81,276.1 (1,25,519.65–2,54,378.31)	8.98 (6.29–12.52)	3,36,957.39 (2,35,303.4–4,68,589.1)	7.66 (5.35–10.66)	−0.72 (−0.78—0.65)
Male	1,15,495.83 (79,647.83–1,62,540.46)	7.22 (5.03–10.12)	2,27,097.7 (1,57,225.88–3,18,773.44)	6.24 (4.33–8.7)	−0.64 (−0.69—0.58)
SDI region					
High SDI	56,549.62 (38,698.89–79,752.38)	5.36 (3.65–7.52)	89,186.24 (61,512.91–1,25,367.71)	4.23 (2.91–5.94)	−0.86 (−0.93—0.79)
High-middle SDI	80,577.72 (55,730.14–1,12,856.78)	8.42 (5.92–11.74)	1,51,425.15 (1,05,795.89–2,10,706.32)	7.47 (5.24–10.37)	−0.51 (−0.6—0.43)
Middle SDI	80,168.16 (54,566.35–1,13,955.76)	8.93 (6.14–12.58)	1,78,884.37 (1,23,116.19–2,52,644.56)	7.62 (5.26–10.74)	−0.83 (−0.96—0.7)
Low-middle SDI	56,343.01 (38,634.82–79,390.34)	10.71 (7.4–15.07)	98,275.14 (67,790.85–1,36,494.68)	7.78 (5.42–10.82)	−1.31 (−1.38—1.24)
Low SDI	23,002.94 (16,015.48–32,345.57)	11.38 (7.98–15.91)	46,042.97 (31,703.49–64,125.98)	10.29 (7.06–14.36)	−0.45 (−0.5—0.41)
South–East Asia, East Asia, and Oceania					
East Asia	55,353.98 (37,834.15–78,429.21)	7.28 (5.02–10.25)	1,43,162.91 (97,844.99–1,98,785.38)	7.01 (4.77–9.78)	−0.53 (−0.8—0.26)
Southeast Asia	20,462.27 (13,700.6–29,390.74)	9.28 (6.26–13.31)	39,747.55 (26,951.3–56,339.11)	7.18 (4.9–10.22)	−1.09 (−1.18—1)
Oceania	86.03 (56.26–125.09)	3.46 (2.33–4.94)	172.19 (113.34–250)	2.95 (1.99–4.26)	−0.4 (−0.44—0.35)
Sub-Saharan Africa					
Eastern Sub-Saharan Africa	9,525.57 (6,372.84–13,595.76)	15.01 (10.11–21.38)	16,275.43 (10,767.18–23,258.98)	11.89 (7.89–16.94)	−0.54 (−0.66—0.42)
Central Sub-Saharan Africa	328.39 (218.34–473.88)	1.94 (1.32–2.79)	780.39 (511.67–1,120.21)	1.99 (1.32–2.85)	0.2 (0.14–0.26)
Southern Sub-Saharan Africa	749.07 (507.68–1,070.84)	3.08 (2.11–4.34)	1,625.79 (1,109.26–2,325.68)	3.29 (2.26–4.7)	0.37 (0.22–0.51)
Western Sub-Saharan Africa	11,611.15 (8,105.96–15,995.84)	14.82 (10.31–20.34)	23,623.94 (16,146.99–32,542.97)	14.57 (10.11–20.1)	−0.35 (−0.48—0.22)
South Asia					
South Asia	61,518.15 (42,096.2–86,249.88)	12.59 (8.75–17.7)	1,10,817.8 (76,989.61–1,54,669.96)	8.54 (5.96–11.93)	−1.75 (−1.87—1.62)
Latin America and Caribbean					
Caribbean	504.5 (337.94–729.04)	2.03 (1.36–2.9)	886.71 (590.77–1,280.88)	1.71 (1.14–2.46)	−0.52 (−0.58—0.45)
Central Latin America	3,855.6 (2,640.4–5,531.32)	5.25 (3.6–7.51)	9,524.43 (6,524.96–13,529.82)	4.21 (2.89–5.97)	−0.71 (−0.77—0.65)
Tropical Latin America	4,137.05 (2,878.72–5,695.31)	5.12 (3.56–7.04)	11,324.86 (7,848.25–15,618.62)	4.82 (3.34–6.68)	−0.09 (−0.25—0.06)
Andean Latin America	1,709.42 (1,157.77–2,427.37)	9.18 (6.25–13.14)	4,391.82 (3,022.64–6,180.43)	8.12 (5.58–11.41)	−0.42 (−0.5—0.34)
North Africa and Middle East					
North Africa and Middle East	25,151.77 (16,785.47–35,942.95)	16.5 (10.96–23.58)	51,536.01 (34,297.27–73,195.78)	13.57 (9.12–19.3)	−0.63 (−0.66—0.6)
Central Europe, Eastern Europe, and Central Asia					
Central Asia	2,186.86 (1,488.98–3,099.7)	5.1 (3.49–7.24)	2,989.07 (2,031.26–4,199.87)	4.73 (3.23–6.57)	−0.31 (−0.41—0.21)
Eastern Europe	6,188.36 (4,124.41–8,842.04)	2.31 (1.56–3.27)	7,150.62 (4,794.29–10,139.43)	2.01 (1.36–2.84)	−0.67 (−0.74—0.61)
Central Europe	6,758.13 (4,613.22–9,511.59)	4.74 (3.24–6.63)	9,434.8 (6,450.88–13,175.46)	4.19 (2.87–5.85)	−0.41 (−0.46—0.37)
High–income regions					
Southern Latin America	1,460.83 (966.34–2,107.65)	3.42 (2.3–4.89)	2,371.84 (1,606.53–3,410.71)	2.76 (1.87–3.95)	−0.68 (−0.7—0.67)
Western Europe	68,785.27 (47,042.98–97,198.86)	11.52 (7.89–16.29)	97,364.81 (67,087.7–1,37,766.58)	9.31 (6.39–13.09)	−0.76 (−0.79—0.73)
North America	10,796.58 (7,397.68–15,397.11)	2.91 (2–4.14)	18,170.68 (12,528.56–25,673.35)	2.64 (1.81–3.75)	−0.45 (−0.6—0.31)
Australasia	1,055.49 (707.49–1,520.13)	4.66 (3.15–6.69)	2,080.78 (1,405.74–2,989.83)	3.86 (2.59–5.58)	−0.63 (−0.69—0.58)
Asia Pacific	4,547.43 (3,014.94–6,477.05)	2.44 (1.63–3.44)	10,622.68 (7,186.98–15,401.48)	2.01 (1.36–2.9)	−0.7 (−0.84—0.55)

AMD, age-related macular degeneration; SDI, socio-demographic index; UI, uncertainty interval; CI, confidence interval; ASR, age-standardized rate; EAPC, estimated annual percentage change; GBD, global burden of disease; YLDs, years lived with disability.

### AMD burden by age

**Figure 2:**
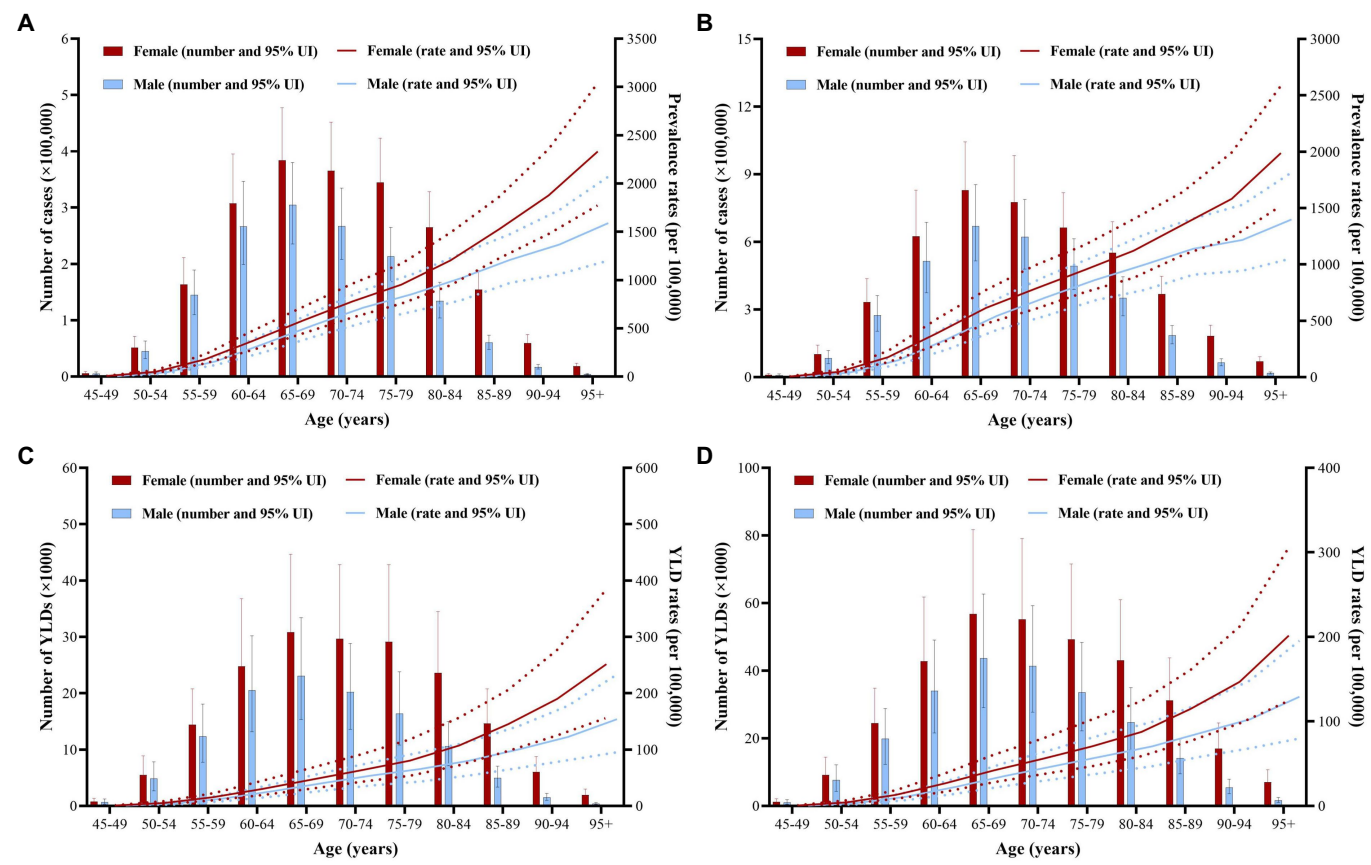
Global burden of the prevalence and YLDs of AMD by age and sex in 1990 and 2019. **(A)** The number of cases and crude prevalence rates of AMD in 1990. **(B)** The number of cases and crude prevalence rates of AMD in 2019. **(C)** The number of YLDs and crude YLD rates of AMD in 1990. **(D)** The number of YLDs and crude YLD rates of AMD in 2019. Red and blue dashed line indicates the upper and lower limits of the 95% uncertainty intervals (95% UIs) for females and males, respectively. AMD, age-related macular degeneration; UI, uncertainty interval; YLDs, years lived with disability.

With age increasing, the global number of cases and YLDs due to AMD gradually increased when age was 50 years old in 1990, reaching the peak at age of 65–69, after that it decreased with age increasing (Figures 2A,C). It has the similar trend in 2019 (Figures 2B,D). The crude prevalence rates and YLD rates of AMD increased with age, the fastest increasing speed is the age over 80 years old in 1990. In 2019, the general trend of the crude prevalence rates and YLD rates is similar to the trend in 1990. Additionally, the rate of increasing of the curves after age of 90 years old showed a significantly increasing in both 1990 and 2019.

### AMD burden by sex

Between 1990 and 2019, the number of AMD cases and YLDs, and ASPRs and ASYRs in females was significantly higher than in males (Tables 1, 2; Figure 1). The female number of AMD cases and YLDs due to AMD were all significantly larger than males in 1990 and 2019, and the difference in crude rates was obviously, especially the age after 80 years old in 1990 and 2019 (Figure 2).

### AMD burden by SDI

**Figure 3:**
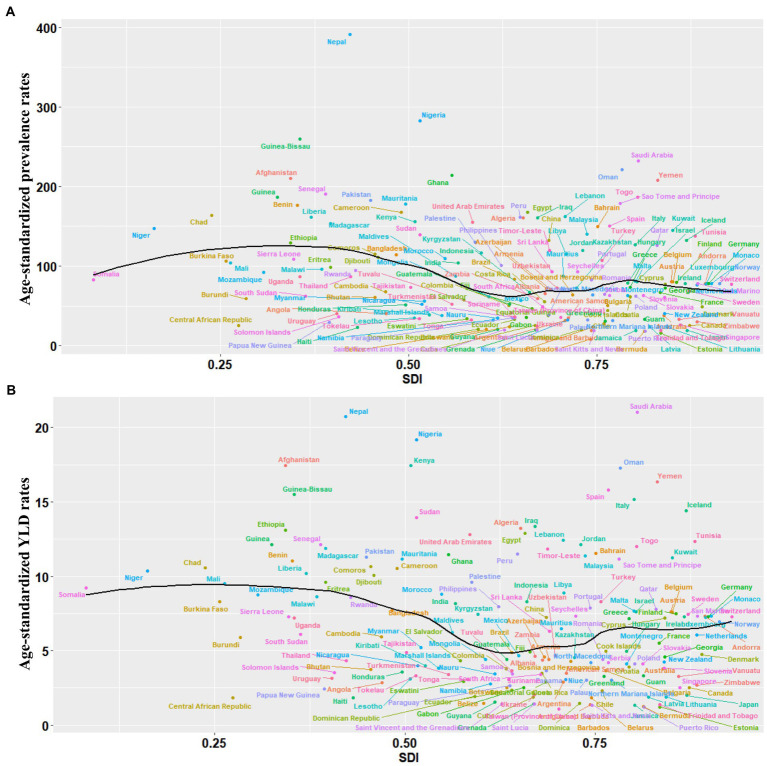
Global burden of the prevalence and YLDs of AMD by SDI. **(A)** The black line represents the average expected relationship between SDIs and age-standardized prevalence rates for AMD based on values from 204 countries and territories in 2019. **(B)** The black line represents the average expected relationship between SDIs and age-standardized YLD rates for AMD based on values from 204 countries and territories in 2019. AMD, age-related macular degeneration; SDI, socio-demographic index; YLDs, years lived with disability.

In 2019, the number of AMD cases and YLDs were the highest in middle SDI regions and the lowest in low SDI regions. Meanwhile, the ASPRs and ASYRs of AMD were highest in the low SDI regions and lowest in the high SDI regions (Tables 1, 2). The relationship between ASPR and ASYR and SDI in 204 countries and territories in 2019 are shown in Figure 3. The relationship between ASPR and ASYR and SDI in 21 regions between 1990 and 2019 are shown in Supplementary Figure S1. ASPRs and ASYRs in developing countries and regions were significantly higher than those in developed countries and regions.

### Regional AMD burden

At the regional level in 1990 and 2019, the number of AMD cases and YLDs were highest in East Asia, followed by South Asia, and lowest in Oceania (Tables 1, 2). The ASPR and ASYR were highest in Western Sub-Saharan Africa, followed by North Africa and Middle East, and lowest in High-income Asia Pacific. From 1990 to 2019, the EAPCs of ASPR and ASYR in South Asia were lowest at −1.41 (95% CI, −1.55 to −1.27) and − 1.75 (95% CI, −1.87 to −1.62), followed by Southeast Asia at −0.75 (95% CI, −0.82 to −0.68) and − 1.09 (95% CI, −1.18 to −1). In contrast, the EAPC of ASPR and ASYR in Southern Sub-Saharan Africa was highest at 0.37 (95% CI, 0.29–0.45) and 0.37 (95% CI, 0.22–0.51), followed by Central Sub-Saharan Africa were 0.26 (95% CI, 0.23–0.29) and 0.2 (95% CI, 0.14–0.26).

### National AMD burden

**Figure 4:**
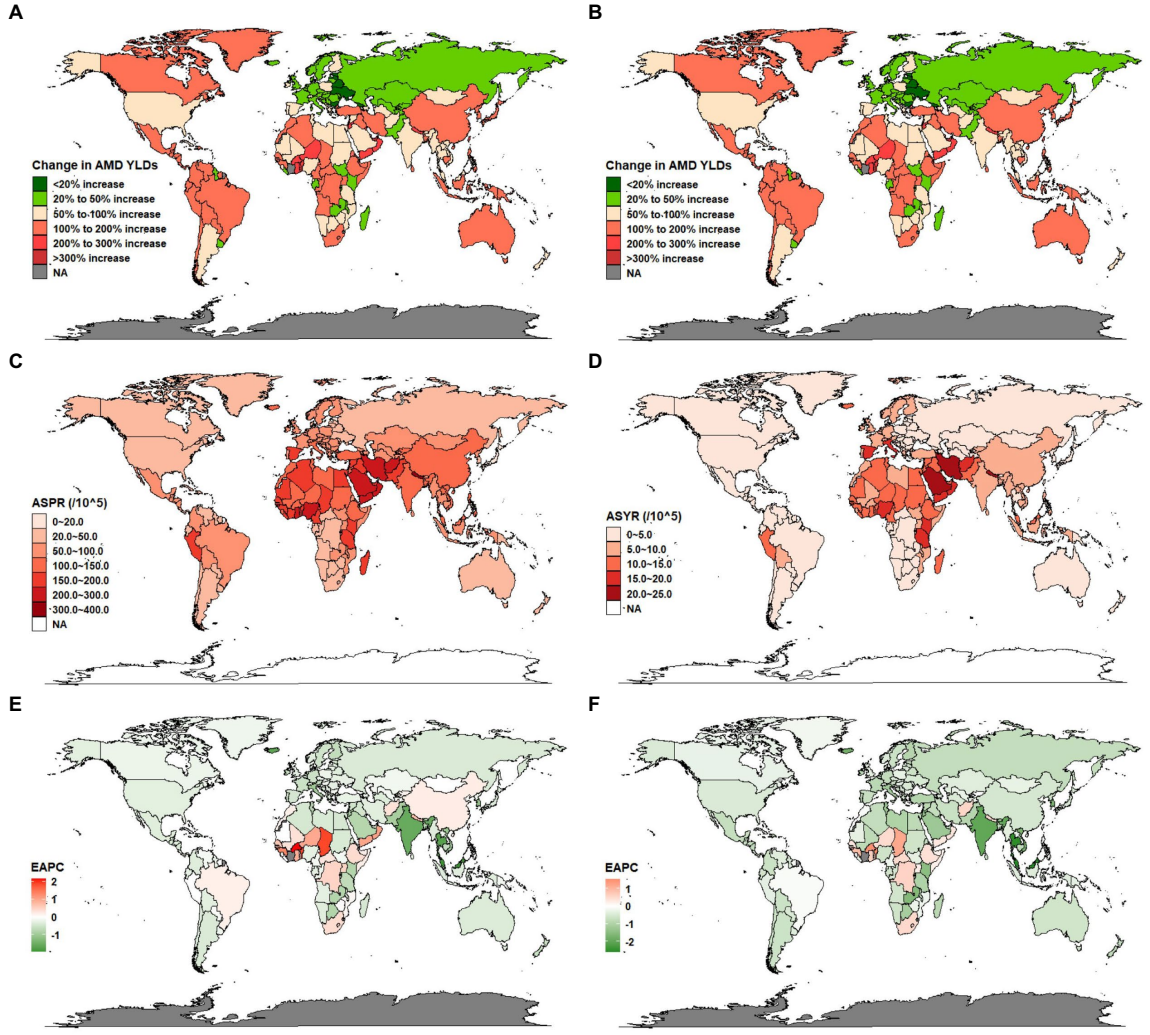
Global burden of the prevalence and YLDs of AMD in 204 countries and territories. **(A)** The growth rates of AMD cases from 1990 to 2019. **(B)** The growth rates of AMD YLDs from 1990 to 2019. **(C)** The ASPR of AMD in 2019. **(D)** The ASYR of AMD in 2019. **(E)** The EAPC of ASPR for AMD from 1990 to 2019. **(F)** The EAPC of ASYR for AMD from 1990 to 2019. AMD, age-related macular degeneration; ASR, age-standardized rate; EAPC, estimated annual percentage change; YLDs, years lived with disability.

The growth rates of cases and YLDs in Burkina Faso, Benin, and Niger were more than 300% increase from 1990 to 2019. While Ukraine, Belarus, Latvia, and Georgia were less than 20% increase during this period (Figures 4A,B). In addition, China and India demonstrated the largest cases and YLDs of AMD in 2019, while Tokelau, Niue and Nauru demonstrated the lowest (Supplementary Tables S1, S2). The ASPRs of AMD in 2019 ranged from 19.1 to 390.9 per 100,000 people in 204 countries and territories, Nepal demonstrated the highest ASPR of AMD, while Barbados demonstrated the lowest (Figure 4C; Supplementary Table S1). The ASYRs in 2019 ranged from 0.51 to 21.6 per 100,000 people in 204 countries and territories, Iran demonstrated the highest ASYR of AMD, while Barbados demonstrated the lowest (Figure 4D; Supplementary Table S2). In addition, Malaysia saw the largest decline in ASPR (EAPC, −1.97; 95% CI, −2.16 to −1.78) and ASYR (EAPC, −2.62; 95% CI, −2.87 to −2.37), followed by Thailand, Iceland, and India. Only a few countries or territories (such as Burkina Faso, Gambia, Chad, Benin, and Côte d’Ivoire) reported an increasing ASPR and ASYR of AMD between 1990 and 2019 (Figures 4E,F; Supplementary Tables S1, S2).

## Discussion

This study reported on the cases and YLDs, and ASPRs and ASYRs of AMD from 1990 to 2019 in 204 countries and territories around the world and revealed disparities by age, sex, socio-economic level, region, and nation. Although the ASPR and ASYR decreased overall from 1990 to 2019 (with a global EAPC of −0.15 and − 0.71), the overall cases and YLDs due to AMD still increased. Furthermore, compared with other age groups, the ASPRs and ASYRs in older adults increased significantly. Globally, aging population is the most important medical and social demographic issue. The global population aged 65 and over in 2000 was 18 percent, while by 2050 it will reach 38 percent (19). The aging population and high prevalence and YLDs of AMD in people over 60 may be the main reasons for the near doubling of overall cases between 1990 and 2019. Due to the age of the global population is increasing, AMD cases will continue to rise.

Global policy making and economic restructuring can guide the prevention and treatment of AMD. Initially, for the purpose of eliminating preventable blindness by 2020, the WHO and the International Agency for the Prevention of Blindness (IAPB) started the VISION 2020 global initiative in 1999 to prevent, control, and eliminate avoidable blindness at the national level (20). In line with earlier studies, the fact that the AMD cases presented a growing tendency globally in the last 30 years, nevertheless, the age-standardized prevalence rates showed a descending trend during this period (13, 21). It indicates that we have made some progress in the fight against AMD, but preventing vision loss caused by AMD remains a major challenge due to population growth and aging.

Although previous studies have implicated many risk factors related to AMD progression, age is uniquely important (22–25). A meta-analysis in 2020, combining data of 26 studies, showed an overall increase in prevalence of AMD in people aged 60^+^ years; the prevalence of early or intermediate AMD increased by 25.3%, and the prevalence of late AMD increased by 2.4%. The combined annual incidence rate of late AMD in all age groups was 1.4/1000 people (26). Another meta-analysis showed that for late AMD, the prevalence rates of people aged 55–59 and 85^+^ years were 0.1 and 9.8%, respectively. For early AMD, the prevalence rates of the two groups were 3.5% and 17.6%, respectively (27). Our study found similar results. The prevalence and YLDs of AMD in the older groups was significantly higher than that of the young group. Aging is the most significant risk factor for AMD because changes in retinal structure and function can promote the development of AMD (28). In time, this may cause other pathological risk factors to have a cumulatively detrimental effect.

The number of AMD cases and YLDs, and the ASPRs and ASYRs were significantly higher in females than in males overall from 1990 to 2019. AMD and its subtypes are equally prevalent in both males and females, and while some studies have shown that females have a faster progression rate than males, this difference was not observed in the more recent 2005–2008 NHANES study (29–31). Sex is often regarded as an independent risk factor, with some studies reporting higher risk in females, while other studies report the opposite (32–35). Possible explanations for the conflicting results include differences in follow-up time points and longer life expectancy in women (36). However, some studies suggested that the progression of AMD may be related to the difference of sex hormones in the body, such as estrogen (37). Another hypothesis is that the difference in AMD progression is related to the number of X-linked genes, which may be associated with the pathogenesis of AMD (38). Further studies may be needed to explore the possible mechanisms between sex and AMD.

Between 1990 and 2019, the ASPRs and ASYRs of low SDI regions were higher than those of high SDI regions. There is no doubt those a higher socio-economic level reduces the burden of disease because in more developed countries, health care systems are better equipped to provide access to preventative medicine, including services for early detection, early diagnosis, and early treatment of diseases (14). In this study, the burden of AMD was significantly different in regions with different socioeconomic statuses. SDI is a summary index that can be used to represent the position of a nation on the spectrum of economic development, and different regions in each nation can have significantly different SDI levels (39). A previous study has reported the AMD burden to be higher in low SDI regions (40). However, this study showed that the middle SDI regions demonstrated the highest number of AMD cases and YLDs, and the low SDI region demonstrated the lowest. The main reason may be that China and India are considered middle SDI regions. In addition, the ASPRs and ASYRs in developing countries and regions were higher than those in developed countries and regions. The burden of AMD in developing countries were higher than those of developed countries. Possible explanations for this phenomenon that the increased levels of ultraviolet radiation in the countries of the South, the prevalence of arterial hypertension, diabetes, et al. maybe contribute to the spread of this pathology (41, 42). Moreover, in response to the VISION 2020, the developed countries funded for various programs to reduce levels of low vision and blindness. Along with more active diagnosis, early detection and treatment could positively influence the performance of the developed countries. More attention and further studies are supposed to be paid to this phenomenon, to explore the possible mechanisms of the disparities.

The prevalence and YLDs of AMD also varied significantly in different regions and nations. From 1990 to 2019, the number of AMD cases and YLDs increased by 117.59 and 90.06%, and the disease burden more than doubled. The ASPR and ASYR of AMD decreased by an EAPC, which may indicate that the increase in overall AMD cases and YLDs are related to the increasing age of the global population, as well as the increasing population. The number of AMD cases and YLDs in East Asia remained highest, followed by South Asia, while the number of AMD cases and YLDs in Oceania remained lowest in 2019. In the past 30 years, East Asia and South Asia have demonstrated increasing cases and YLDs of AMD, primarily because of the population sizes and land area of China and India. However, the ASPR and ASYR in East Asia demonstrated an increasing trend, while the ASPR and ASYR in South Asia demonstrated a decreasing trend. This discrepancy may be due to the different population structures and average ages of the major countries in the two regions. The population of India is also growing much faster than the population of China; however, the proportion of older adults (older than 65 years) in China is far higher than in India because China experienced population aging earlier than India (43–45). One meta-analysis of Chinese populations has indicated that, as a result of the rapid population aging, the total prevalent AMD cases and its subtypes has increased dramatically: from 1990 to 2015, cases of AMD had increased by 121.80% in China (46). The social security system of China and India should be improved so that older adults have the basic financial security they need to reduce the burden of AMD.

Between 1990 and 2019, the EAPCs of ASPR and ASYR in Southern Sub-Saharan Africa were 0.37 (the highest among all regions), and the number of cases and YLDs in this region increased significantly in the past 30 years. This region has unique characteristics, including the highest population growth rate and high rates of poor living conditions and poor health; the average life expectancy of people born in this region is only 54 years (47). Compared with other regions of the world, the burden of non-communicable and communicable diseases in this region is significantly heavier (48). The overall level of social, economic, and educational development in this region is low, which represents a microcosm of many low and low-middle SDI regions. Additionally, because many people still live in tribal societies, most countries of Southern Sub-Saharan Africa are highly underdeveloped (49). Thus, in developing countries, it is as important as developed counties to emphasize the priority of reducing AMD burden when making health policy.

## Limitations

Although the 2019 GBD study enables us to study the global AMD burden with high confidence, some limitations should be noted. First, as noted by other research reports of the GBD study, actual data on disease burden cannot be obtained, only estimated. Second, the nature of the database makes differences inevitable in data collection methods and data source quality between studies. Thirdly, because of the lack of relevant data in the GBD database, prevalence over time based on specific characteristics of AMD, such as dry (non-neovascular) versus wet (neovascular), could not be assessed in this study. Moreover, the incidence of AMD was not examined in this study.

## Conclusion

Overall, despite reductions in ASPR and ASYR, with the global aged population increasing, AMD remains a major cause of irreversible vision loss and burden across the world. The prevalent AMD cases and YLDs, and the ASPR and ASYR due to AMD were higher in females than in males. The prevalence and YLDs of AMD were also closely related to socio-economic status, and they varied geographically, especially people in less developed countries seemed to be more likely to bear heavier burden of AMD. Knowledge of differences in development levels of countries and how these differences affect the prevalence and YLDs of AMD could help policy-makers and public health specialists formulate better approaches to treatment and prevention. These findings can help us better understand the global disease burden of AMD, and the influences of the included factors may provide a theoretical basis for governments and health care planners to establish more effective and targeted AMD prevention strategies.

## Data availability statement

The original contributions presented in the study are included in the article/Supplementary material, further inquiries can be directed to the corresponding author.

## Author contributions

PL made contributions to the conception and design of the work, provided administrative support, and analyzed the data in our study. BJ collected and organized the data, made contributions to the acquisition, analysis and interpretation of data, and was a major contributor in writing the manuscript. JL made contributions to the critical revision of the manuscript for important intellectual content. CJ made contributions to writing the manuscript. All authors read and approved the final manuscript.

## Funding

This study was supported by Jiangsu Provincial Medical Innovation Team (grant No. CXTDA2017039) and the National Natural Science Foundation in China (grant No. 81671641, 82271113) to PL.

## Conflict of interest

The authors declare that the research was conducted in the absence of any commercial or financial relationships that could be construed as a potential conflict of interest.

## Publisher’s note

All claims expressed in this article are solely those of the authors and do not necessarily represent those of their affiliated organizations, or those of the publisher, the editors and the reviewers. Any product that may be evaluated in this article, or claim that may be made by its manufacturer, is not guaranteed or endorsed by the publisher.
