# Supplementary material for: Trends and disparities in disease burden of age-related macular degeneration from 1990 to 2019: Results from the global burden of disease study 2019
Source: Front Public Health. 2023 Apr 17;11:1138428. doi: 10.3389/fpubh.2023.1138428 (PMC10231224; doi:10.3389/fpubh.2023.1138428)
Supplement: Supplementary file 1 [file Table_1.doc]

**Table S1. Estimated number and age-standardized rate (per 100,000 persons) of prevalence and temporal trends for AMD in 204 countries and territories from 1990 to 2019.**

| **Country** | **1990** | | **2019** | | **1990-2019** |
| --- | --- | --- | --- | --- | --- |
|  | **Number (95% UI)** | **ASR (95% UI)** | **Number (95% UI)** | **ASR (95% UI)** | **EAPC (95% CI)** |
| Afghanistan | 13552.49 (10882.59-16577.41) | 202.3 (165.17-245.67) | 19446.54 (15900.2-23495.04) | 210.12 (172.06-253.26) | 0.31 (0.23-0.39) |
| Albania | 1301.55 (1054.5-1572.93) | 70.6 (57.8-84.89) | 2890.12 (2334.77-3513.4) | 65.11 (52.78-78.88) | -0.31 (-0.34--0.28) |
| Algeria | 21135.73 (16952.69-25813.84) | 185.37 (150.96-223.26) | 49346.32 (39699.44-60274.71) | 160.55 (130.71-194.22) | -0.42 (-0.44--0.4) |
| American Samoa | 7.41 (5.81-9.22) | 39.05 (30.98-47.92) | 15.33 (12.17-18.87) | 34.95 (27.9-42.53) | -0.31 (-0.35--0.28) |
| Andorra | 38.89 (32.01-46.06) | 85.49 (71.49-100.2) | 116.14 (97.18-136.53) | 77.71 (64.52-91.61) | -0.34 (-0.37--0.3) |
| Angola | 1205.78 (942.12-1503.62) | 42.79 (33.97-52.5) | 3264.91 (2547.87-4050.62) | 39.98 (31.72-49.17) | -0.27 (-0.3--0.24) |
| Antigua and Barbuda | 11.66 (9.21-14.34) | 20.77 (16.47-25.62) | 18.33 (14.54-22.68) | 19.34 (15.3-24.02) | -0.21 (-0.24--0.19) |
| Argentina | 10917.12 (8646.88-13332.16) | 36.13 (29.12-43.68) | 17724.75 (14170.89-21485.92) | 31.78 (25.42-38.5) | -0.39 (-0.41--0.37) |
| Armenia | 1745.29 (1406.01-2140.2) | 71.75 (57.44-87.09) | 2980.36 (2371.3-3656.97) | 70.58 (56.05-85.93) | -0.15 (-0.19--0.11) |
| Australia | 7915.86 (6471.03-9389.86) | 41.97 (34.73-49.8) | 17157.08 (14114.58-20562.72) | 37.5 (30.76-44.9) | -0.36 (-0.44--0.27) |
| Austria | 11606.05 (9646.41-13704.69) | 93.35 (78.13-109.66) | 16316.97 (13680.93-19177.27) | 80.48 (67.23-94.23) | -0.53 (-0.55--0.51) |
| Azerbaijan | 3308.16 (2652.97-4017.19) | 73.83 (58.92-89.37) | 5731.6 (4487.65-7173.18) | 71.46 (57.23-87.04) | -0.2 (-0.25--0.15) |
| Bahamas | 28.49 (22.78-34.96) | 20.82 (16.6-25.39) | 68.84 (54.82-85.56) | 19.53 (15.5-24.11) | -0.18 (-0.22--0.14) |
| Bahrain | 242.37 (194.66-294.61) | 178.06 (143.58-214.46) | 1141.51 (894.66-1409.07) | 149.49 (120.83-180.52) | -0.58 (-0.6--0.56) |
| Bangladesh | 57691.85 (46242.92-70530.6) | 139.9 (113.11-169.88) | 141538.62 (115443.88-171155.18) | 113.96 (93.29-136.94) | -0.7 (-0.79--0.6) |
| Barbados | 26.81 (20.83-33.86) | 8.46 (6.62-10.51) | 41.74 (32.55-52.78) | 8.25 (6.46-10.39) | 0 (-0.03-0.03) |
| Belarus | 3286.1 (2633.83-4041.59) | 25.57 (20.51-31.37) | 3889.04 (3108.76-4722.13) | 23.57 (18.81-28.6) | -0.32 (-0.35--0.29) |
| Belgium | 14708.57 (12186.66-17354.27) | 92.14 (76.93-108.43) | 21161.25 (17623.47-25084.12) | 80.53 (67.01-94.92) | -0.49 (-0.51--0.48) |
| Belize | 19.52 (15.65-23.81) | 21.98 (17.66-26.79) | 49.06 (39.44-60.17) | 20.25 (16.24-24.98) | -0.24 (-0.27--0.21) |
| Benin | 1764.88 (1433.44-2129.81) | 97.95 (79.74-117.63) | 7411.92 (5857.84-9216.2) | 176.15 (139.72-215.68) | 1.86 (1.68-2.04) |
| Bermuda | 12.06 (9.6-14.96) | 20.18 (16.11-24.89) | 25.23 (19.96-31.36) | 18.39 (14.54-22.82) | -0.28 (-0.31--0.25) |
| Bhutan | 150.65 (120.81-186.38) | 77.49 (63.5-94.37) | 308.13 (246.46-384.34) | 60.35 (48.39-74.78) | -0.95 (-1.14--0.76) |
| Bolivia (Plurinational State of) | 2381.2 (1932.98-2892.89) | 83.82 (68.3-100.63) | 6280.1 (5054.7-7660.52) | 75.86 (61.69-91.92) | -0.3 (-0.32--0.28) |
| Bosnia and Herzegovina | 2461.13 (1973.5-3002.74) | 69.77 (56.29-84.37) | 3916 (3151.01-4824.17) | 64.15 (51.81-78.77) | -0.4 (-0.46--0.34) |
| Botswana | 139.77 (103.53-180.98) | 29.77 (22.72-37.71) | 257.65 (193.49-329.5) | 23.98 (18.54-30.11) | -0.79 (-0.84--0.74) |
| Brazil | 67652.66 (55606.25-81197.17) | 85.05 (70.21-101) | 191787.77 (159365.82-228780.09) | 83.13 (68.84-99.18) | 0.17 (0.01-0.34) |
| Brunei Darussalam | 14.18 (11.1-17.63) | 24.04 (19.12-29.46) | 42.64 (33.33-53.07) | 22.79 (18.1-27.74) | -0.15 (-0.22--0.09) |
| Bulgaria | 6369.89 (5396.16-7386.74) | 53.22 (45.41-61.23) | 7874.86 (6317.83-9563.56) | 50.4 (40.8-60.87) | -0.15 (-0.18--0.12) |
| Burkina Faso | 1815.47 (1475.12-2193.05) | 49.37 (40.21-59.16) | 8343.04 (6669.14-10269.38) | 106.03 (86.09-129.57) | 2.15 (1.77-2.53) |
| Burundi | 1371.2 (1103.01-1658.3) | 67.76 (54.78-81.31) | 2122.37 (1744.83-2543.67) | 58.99 (48.23-70.56) | -0.49 (-0.54--0.45) |
| Cabo Verde | 517.28 (444.88-601.36) | 220.25 (189.83-256.06) | 760.08 (601.17-965.67) | 191.5 (150.49-243.89) | -0.56 (-0.61--0.51) |
| Cambodia | 3218.21 (2484.64-4033.81) | 83.19 (65.02-103.51) | 7248.52 (5510.24-9134.91) | 67.53 (52.02-84.3) | -0.66 (-0.77--0.55) |
| Cameroon | 6254.04 (4966.36-7678.55) | 164.59 (131.45-200.53) | 16698.84 (13250.16-20651.31) | 167.51 (133.63-205.75) | -0.11 (-0.24-0.01) |
| Canada | 8603.37 (7019.67-10319.98) | 26.82 (21.98-32.16) | 19252.01 (15790.87-23055.71) | 25.55 (20.93-30.51) | -0.19 (-0.21--0.18) |
| Central African Republic | 175.08 (138.55-218.09) | 23.36 (18.88-28.48) | 330.69 (262.78-404.88) | 25.12 (20.29-30.62) | 0.29 (0.27-0.31) |
| Chad | 2608.29 (2145.85-3100.43) | 99.44 (82.21-117.4) | 7853.18 (6304.92-9685.5) | 163.77 (132.16-200.06) | 1.87 (1.65-2.1) |
| Chile | 2680 (2112.6-3263.17) | 30.18 (23.99-36.84) | 6665.19 (5304.14-8106.45) | 27.56 (21.99-33.56) | -0.38 (-0.4--0.36) |
| China | 887438.63 (721285.68-1065956.29) | 121.33 (100.83-144.26) | 2601883.03 (2134011.4-3114761.87) | 131.97 (109.37-156.91) | 0.21 (0.05-0.36) |
| Colombia | 8544.01 (6866.14-10326.22) | 56.14 (45.17-67.65) | 26945.77 (21721.65-32657.65) | 50.62 (40.64-61.66) | -0.33 (-0.35--0.3) |
| Comoros | 270.25 (216.4-327.94) | 133.3 (107.72-160.8) | 511.43 (414.66-615.77) | 114.97 (93.28-138.33) | -0.53 (-0.55--0.51) |
| Congo | 407.01 (312.67-512.61) | 47.61 (37.86-58.64) | 916.25 (710.82-1153.43) | 46.28 (36.44-57.26) | -0.15 (-0.18--0.12) |
| Cook Islands | 6.47 (4.96-8.22) | 55.75 (43.15-70.07) | 11 (8.55-13.73) | 43.88 (34.16-54.75) | -0.6 (-0.68--0.51) |
| Costa Rica | 939.14 (755.02-1138.09) | 57.55 (46.42-69.77) | 2809.84 (2278.14-3401.1) | 55.32 (44.57-67.16) | -0.13 (-0.15--0.11) |
| Côte d'Ivoire | 1964.46 (1571.53-2417.99) | 69.2 (55.97-82.48) | 16296.48 (12898.89-20357.88) | 184.61 (148.16-225.79) | 1.68 (0.98-2.39) |
| Croatia | 4010.21 (3205.09-4918.48) | 64.66 (52.03-78.48) | 5787.81 (4596.57-7160.62) | 61.71 (49.1-75.85) | -0.2 (-0.23--0.17) |
| Cuba | 2756.05 (2190.61-3396.89) | 26.99 (21.49-33.2) | 4966.42 (3969.08-6068.12) | 25.29 (20.18-30.81) | -0.16 (-0.19--0.12) |
| Cyprus | 629.53 (510.73-752.83) | 88.46 (73.72-104.73) | 1453.14 (1181.55-1731.55) | 77.55 (64.34-91.73) | -0.48 (-0.5--0.45) |
| Czechia | 9001.07 (7239.29-10962.67) | 64.47 (52.29-77.78) | 13600.45 (10908.24-16771.47) | 60.98 (48.97-74.36) | -0.2 (-0.23--0.16) |
| Democratic People's Republic of Korea | 7377.18 (5710.15-9302.23) | 58.34 (45.79-73.68) | 16953.11 (13367.49-21449.26) | 56.15 (44.47-70.85) | -0.16 (-0.19--0.13) |
| Democratic Republic of the Congo | 2876.09 (2219.64-3623.56) | 24.45 (19.26-30.14) | 7069.43 (5595.56-8726.46) | 27.23 (21.79-33.2) | 0.47 (0.41-0.53) |
| Denmark | 4759.26 (3948.21-5654.74) | 54.71 (45.75-64.53) | 6211.87 (5089.18-7369.16) | 48.93 (40.23-58.09) | -0.39 (-0.42--0.36) |
| Djibouti | 138.16 (110.47-167.1) | 130.11 (105.72-157.12) | 530.5 (427.92-649.43) | 111.19 (90.64-134.04) | -0.6 (-0.64--0.56) |
| Dominica | 16.16 (12.75-20.06) | 21.1 (16.75-25.93) | 18.38 (14.6-22.64) | 19.91 (15.83-24.5) | -0.15 (-0.19--0.11) |
| Dominican Republic | 805.62 (633.4-1012.88) | 24.35 (19.17-30.19) | 1814.42 (1423.43-2237.38) | 20.78 (16.31-25.71) | -0.45 (-0.5--0.4) |
| Ecuador | 1751.09 (1410.74-2108.26) | 36.07 (29.02-43.45) | 4788.69 (3817.47-5847.14) | 32.95 (26.42-40.03) | -0.26 (-0.28--0.24) |
| Egypt | 49599.38 (40169.01-59985.8) | 194.91 (159.39-234.38) | 93178.11 (74887.21-113817.81) | 167.46 (135.96-201.59) | -0.43 (-0.46--0.4) |
| El Salvador | 1831.33 (1462.56-2236.62) | 66.39 (53.06-81.01) | 3450.61 (2728.62-4259.1) | 57.14 (45.08-70.94) | -0.37 (-0.44--0.31) |
| Equatorial Guinea | 66.87 (52.58-82.86) | 42.89 (34.35-52.19) | 142.34 (112.01-175.48) | 38.19 (30.36-46.44) | -0.5 (-0.56--0.44) |
| Eritrea | 749.62 (596.04-941.73) | 105.28 (85.18-129.15) | 2007.51 (1586.85-2470.72) | 98.53 (78.59-121.42) | -0.36 (-0.48--0.25) |
| Estonia | 351.53 (277.51-430.51) | 17.17 (13.64-20.92) | 453.59 (357.98-555.36) | 15.5 (12.28-18.85) | -0.45 (-0.48--0.42) |
| Eswatini | 81.91 (64.58-100.58) | 34.98 (27.75-42.47) | 158.01 (124.83-195.49) | 33.61 (26.98-40.76) | -0.13 (-0.17--0.09) |
| Ethiopia | 19271.77 (16033.54-23143.57) | 115.64 (97.58-137.2) | 46309.91 (38403.02-55690.58) | 128.84 (107.25-154.19) | 0.39 (0.27-0.52) |
| Fiji | 138.33 (107.36-174.03) | 48.17 (38.27-59.8) | 298.99 (240.85-367.5) | 46.42 (37.4-56.15) | 0.02 (-0.06-0.09) |
| Finland | 6765.35 (5611.79-8004.8) | 94.37 (78.77-111.24) | 11144.59 (9247.21-13117.88) | 79.91 (66.2-93.68) | -0.62 (-0.66--0.59) |
| France | 69052.24 (56592.05-83023.35) | 77.26 (63.71-91.83) | 107833.96 (88498.86-129274.59) | 66.71 (54.83-80.17) | -0.54 (-0.57--0.51) |
| Gabon | 185.65 (147.52-229.97) | 38.35 (30.6-46.82) | 298.75 (232.01-368.92) | 35.31 (27.64-43.12) | -0.29 (-0.31--0.27) |
| Gambia | 289.81 (236.82-350.21) | 95.25 (78.35-114.17) | 1360.03 (1072.42-1690.96) | 159.55 (126.95-198.01) | 1.94 (1.77-2.11) |
| Georgia | 4238.7 (3391.07-5231.96) | 71.32 (57.06-87.62) | 4595.58 (3695.22-5580.56) | 72.36 (58.2-88.24) | 0.02 (0-0.05) |
| Germany | 121176.85 (100707.45-142602.06) | 91.52 (76.46-107.16) | 171892.93 (142118.88-204087.9) | 78.11 (65.15-91.88) | -0.53 (-0.57--0.49) |
| Ghana | 7958.06 (6331.56-9906.36) | 152.02 (122.79-187.17) | 30401.78 (24019.54-37937.85) | 213.76 (171.42-262.71) | 0.88 (0.61-1.16) |
| Greece | 12468.25 (10276.37-14839.88) | 82.92 (68.86-98.31) | 20667.89 (16984.27-24690.25) | 73.09 (60.71-86.45) | -0.45 (-0.49--0.41) |
| Greenland | 7.56 (6.1-9.05) | 32.49 (26.51-39.08) | 17.41 (14.19-21.02) | 30.63 (25.09-36.89) | -0.17 (-0.19--0.15) |
| Grenada | 17.01 (13.55-20.72) | 21.67 (17.35-26.24) | 20.32 (16.01-25.38) | 19.59 (15.55-24.29) | -0.29 (-0.34--0.23) |
| Guam | 23.18 (18.23-28.94) | 36.47 (29.21-44.44) | 62.05 (49.66-76.22) | 33.14 (26.45-40.69) | -0.2 (-0.25--0.14) |
| Guatemala | 1828.58 (1391.37-2323.91) | 60.26 (46.32-75.72) | 5360.46 (4130.47-6790.6) | 51.64 (40.07-65.15) | -0.39 (-0.48--0.3) |
| Guinea | 3748.14 (2991.39-4586.55) | 121.01 (97.15-146.67) | 9292.66 (7399.48-11301.38) | 186.21 (148.54-225.46) | 1.31 (1.13-1.49) |
| Guinea-Bissau | 563.29 (446.79-707.62) | 161.6 (129.39-200) | 1558.84 (1227.22-1955.04) | 259.8 (207.62-320.04) | 1.3 (1.06-1.54) |
| Guyana | 72.74 (57.8-89.36) | 22.38 (17.92-27.42) | 110.91 (87.89-137.57) | 20.48 (16.2-25.12) | -0.22 (-0.26--0.18) |
| Haiti | 660.82 (523.37-812.36) | 24.59 (19.63-29.93) | 1307.64 (1034.65-1605.01) | 22.67 (18.03-27.52) | -0.23 (-0.26--0.21) |
| Honduras | 1014.37 (802.2-1266.45) | 56.45 (44.47-70.2) | 2737.6 (2126.77-3468.7) | 51.05 (39.61-63.78) | -0.32 (-0.35--0.29) |
| Hungary | 12132.79 (9809.92-14740.04) | 81.23 (65.98-98.33) | 16056.36 (13024.62-19477.26) | 78.42 (64.04-95.67) | -0.15 (-0.2--0.11) |
| Iceland | 586.58 (500.35-679.28) | 197.79 (168.92-228.16) | 806.14 (676.24-945.72) | 132.08 (110.54-155.98) | -1.68 (-1.87--1.5) |
| India | 545122.25 (441424.72-662739.01) | 141.85 (116.78-169.04) | 1100928.46 (897125.32-1330565.36) | 103.55 (84.86-123.81) | -1.57 (-1.76--1.37) |
| Indonesia | 60515.89 (46854.71-76080.76) | 74.07 (58.23-92) | 125501.87 (96844.28-156534.47) | 67.24 (52.41-83.91) | -0.31 (-0.39--0.23) |
| Iran (Islamic Republic of) | 70358.06 (57692.87-84178.26) | 315.98 (265.58-371.31) | 181754.61 (152228.39-212936.1) | 277.18 (232.99-323.69) | -0.3 (-0.38--0.22) |
| Iraq | 12842.2 (10382.8-15519) | 184.5 (150.21-221.25) | 31223.42 (25345.05-37895.58) | 160.92 (131.92-193.03) | -0.46 (-0.5--0.41) |
| Ireland | 3673.46 (3014.28-4349.19) | 92.1 (76.79-108.53) | 6056.08 (5058.05-7133.37) | 77.94 (65.07-91.58) | -0.61 (-0.63--0.59) |
| Israel | 4338.44 (3559.62-5176.39) | 92.4 (77.04-109.57) | 9921.97 (8236.72-11699.23) | 80.2 (66.66-94.52) | -0.5 (-0.54--0.46) |
| Italy | 150196.27 (124475.3-177861.6) | 167.11 (139.52-197.34) | 214684.85 (179817.1-251957.42) | 127.03 (107.1-148.46) | -1.01 (-1.1--0.91) |
| Jamaica | 385.23 (306.21-472.56) | 20.97 (16.81-25.69) | 581.26 (462.62-716.33) | 19.28 (15.3-23.81) | -0.26 (-0.3--0.23) |
| Japan | 33810.41 (27670.29-40348.87) | 21.12 (17.49-25.21) | 85111.63 (70623.31-101100.99) | 19.17 (15.89-22.73) | -0.35 (-0.45--0.24) |
| Jordan | 1621.55 (1268.06-2007.91) | 155.33 (121.84-192.12) | 6313.65 (5109-7653.09) | 119.61 (97.18-144.2) | -0.91 (-0.95--0.87) |
| Kazakhstan | 8562.32 (6898.24-10609.03) | 74.42 (60.18-91.59) | 11437.51 (9086.01-14163.81) | 72.8 (58.01-89.13) | -0.14 (-0.19--0.09) |
| Kenya | 17884.44 (13716.86-22515.63) | 248.55 (192.13-311.02) | 28822.69 (23241.51-35060.75) | 155.64 (125.97-188.41) | -0.8 (-1.1--0.49) |
| Kiribati | 13.07 (10.14-16.59) | 43.3 (34.42-53.61) | 21.3 (16.37-26.81) | 39 (31-47.93) | -0.31 (-0.33--0.28) |
| Kuwait | 733.39 (591.37-883.91) | 163.1 (132.08-195.13) | 2825.27 (2275.78-3459.7) | 144.69 (115.98-175.27) | -0.37 (-0.4--0.35) |
| Kyrgyzstan | 3255.99 (2626.01-4016.52) | 112.28 (90.2-137.64) | 4636.11 (3721.37-5690.19) | 116.49 (94.02-141.13) | 0.16 (0.13-0.19) |
| Lao People's Democratic Republic | 2601.76 (2067.08-3211.39) | 146.34 (119.02-179.7) | 5318.45 (4240.51-6576.98) | 143.97 (115.76-176.35) | -0.19 (-0.26--0.12) |
| Latvia | 918.99 (733.33-1118.79) | 25.41 (20.36-30.98) | 1034.87 (813.49-1271.18) | 23.5 (18.52-28.68) | -0.34 (-0.37--0.31) |
| Lebanon | 3788.55 (3169.13-4456.58) | 177.88 (150.6-207.67) | 8406.96 (6840.76-10068.46) | 162.26 (131.63-194.94) | -0.08 (-0.18-0.02) |
| Lesotho | 306.98 (242-376.22) | 35.74 (28.69-43.33) | 366.99 (288.41-460.65) | 34.91 (27.86-42.83) | -0.05 (-0.09--0.02) |
| Liberia | 1705.67 (1370.55-2111.18) | 160.69 (129.92-196.32) | 2708.62 (2170.11-3353.27) | 161.06 (130-195.87) | -0.02 (-0.04-0.01) |
| Libya | 2266.86 (1833.85-2746.02) | 137.95 (111.52-166.86) | 4947.94 (4039.57-6025.74) | 114.75 (93.71-138.96) | -0.4 (-0.46--0.33) |
| Lithuania | 1135.42 (908.62-1375.51) | 24.93 (20.02-30.18) | 1499.77 (1193.65-1835.35) | 23.67 (18.94-29.03) | -0.23 (-0.24--0.21) |
| Luxembourg | 497.67 (409.07-589.9) | 90.98 (75.61-107.02) | 847.67 (704.09-995.88) | 77.95 (64.87-91.56) | -0.56 (-0.59--0.53) |
| Madagascar | 7757.32 (6334.08-9371.53) | 173.08 (141.93-207.35) | 13080.54 (10551.48-16047.19) | 153.79 (125.02-185.3) | -0.42 (-0.47--0.37) |
| Malawi | 3731.7 (2921.12-4595.39) | 112.64 (89.45-136.64) | 5971.44 (4791.82-7205.55) | 95.98 (77.29-114.89) | -0.37 (-0.46--0.27) |
| Malaysia | 18049.43 (14577.72-21832.39) | 220.53 (179.18-265.84) | 35546.32 (28747.6-43397.86) | 139.92 (113.33-169.39) | -1.97 (-2.16--1.78) |
| Maldives | 71.41 (54.66-91.45) | 102.68 (80-128.63) | 217.24 (167.33-278.03) | 87.24 (66.88-111.79) | -0.62 (-0.7--0.55) |
| Mali | 3445.5 (2960.63-3980.54) | 93.91 (81.13-108.04) | 7919.97 (6410.08-9656.11) | 103.92 (84.92-124.89) | 0.33 (0.19-0.47) |
| Malta | 365.98 (299.82-435.93) | 91.69 (75.93-108.82) | 818.37 (677.79-967.16) | 80.77 (66.94-95.37) | -0.45 (-0.47--0.43) |
| Marshall Islands | 5.94 (4.64-7.43) | 42.92 (34.16-52.76) | 10.46 (8.09-13.15) | 37.73 (29.96-46.29) | -0.42 (-0.45--0.38) |
| Mauritania | 1684.16 (1353.83-2084.52) | 179.81 (145.76-220.51) | 3423.47 (2725.54-4237.26) | 177.75 (142.51-218.37) | -0.01 (-0.03-0.01) |
| Mauritius | 674.46 (545.03-816.74) | 100.9 (82.27-121.48) | 1520.59 (1232.42-1844.46) | 87.75 (71.16-106.05) | -0.51 (-0.52--0.49) |
| Mexico | 24159.86 (19923.79-28948.02) | 64.25 (53.2-76.6) | 62394.25 (51381.92-73982.24) | 56.63 (46.68-67.03) | -0.32 (-0.36--0.29) |
| Micronesia (Federated States of) | 17.32 (13.35-21.68) | 43.03 (33.74-52.94) | 21.2 (16.48-26.69) | 36.59 (28.9-45.14) | -0.49 (-0.52--0.46) |
| Monaco | 69.32 (57.22-81.7) | 85.59 (71.41-100.38) | 86.61 (71.6-101.99) | 77.99 (64.65-91.84) | -0.35 (-0.37--0.32) |
| Mongolia | 579.19 (441.49-745.97) | 63.06 (47.86-80.13) | 1096.69 (833.39-1399.9) | 61.59 (47.17-77.82) | -0.01 (-0.05-0.04) |
| Montenegro | 381.56 (310.08-465.13) | 64.59 (52.7-78.14) | 627.17 (502.23-763.35) | 62.63 (50.46-75.82) | -0.15 (-0.18--0.12) |
| Morocco | 13721.75 (12308.11-15350.37) | 109.47 (98.83-122.1) | 31151.35 (24751.03-37888.78) | 109.36 (87.92-131.28) | 0.18 (0.06-0.31) |
| Mozambique | 4663.74 (3634.61-5846.57) | 92.16 (72.32-114.34) | 8343.01 (6540.79-10462.02) | 92.41 (73.08-114.72) | 0.09 (0.03-0.16) |
| Myanmar | 12459.89 (9886.7-15209.28) | 61.59 (49.71-74.94) | 23471.23 (18457.16-28818.15) | 55.92 (44.63-68.73) | -0.33 (-0.54--0.12) |
| Namibia | 224.98 (176.46-280.35) | 35.14 (28.15-42.84) | 394.59 (312.61-485.66) | 32.26 (25.67-39.24) | -0.32 (-0.35--0.29) |
| Nauru | 1.04 (0.81-1.31) | 38.96 (31.09-47.97) | 0.97 (0.75-1.23) | 34.84 (27.7-42.3) | -0.27 (-0.31--0.24) |
| Nepal | 24017.04 (18958.45-30044.96) | 294.93 (235.09-362.36) | 83237.57 (67095.53-101242.9) | 390.86 (318.08-470.11) | 0.73 (0.57-0.89) |
| Netherlands | 17897.36 (14733.53-21414.08) | 86.9 (71.7-103.29) | 28277.92 (23293.43-33857.4) | 76.11 (62.78-90.95) | -0.5 (-0.53--0.48) |
| New Zealand | 1839.09 (1508.5-2202.36) | 47.61 (39.4-56.61) | 3436.47 (2884.55-4063.5) | 40.44 (33.85-47.74) | -0.56 (-0.59--0.53) |
| Nicaragua | 772.48 (625.06-932.81) | 58.23 (47.06-70.07) | 2041.41 (1638.85-2484.82) | 52.14 (41.93-63.07) | -0.39 (-0.41--0.37) |
| Niger | 2386.61 (1964.32-2858.71) | 102.97 (85.08-121.92) | 9935.67 (7856.35-12393.03) | 147.3 (117.98-180.35) | 0.95 (0.73-1.16) |
| Nigeria | 121687.14 (99570.83-146022.06) | 299.38 (247.27-355.18) | 213421.34 (173009.55-257872.89) | 282.25 (230.84-338.19) | -0.25 (-0.4--0.1) |
| Niue | 0.87 (0.7-1.06) | 38.4 (30.56-47.27) | 0.7 (0.56-0.86) | 32.39 (25.93-39.39) | -0.55 (-0.59--0.51) |
| North Macedonia | 1156.24 (933.05-1406.58) | 68.46 (55.3-82.68) | 1996.06 (1590.19-2451.18) | 63.88 (51.87-77.64) | -0.28 (-0.31--0.24) |
| Northern Mariana Islands | 4.19 (3.27-5.28) | 35.45 (28.43-43.29) | 14.66 (11.45-18.41) | 31.86 (25.44-39.03) | -0.23 (-0.29--0.16) |
| Norway | 6289.27 (5390.71-7320.67) | 84.35 (72.42-97.48) | 8310.22 (7097.66-9644.54) | 78.2 (66.58-90.87) | -0.31 (-0.35--0.27) |
| Oman | 749.13 (605.02-926.21) | 139.28 (113.93-170.16) | 2939.64 (2354-3605.74) | 221.28 (179.62-265.75) | 1.03 (0.56-1.49) |
| Pakistan | 119823.67 (97741.18-146170.47) | 227.89 (187.23-276.38) | 173071.43 (139394.41-210371.63) | 182.37 (149.82-220.88) | -0.91 (-1.01--0.8) |
| Palau | 3.22 (2.53-3.99) | 37.05 (29.43-45.31) | 5.93 (4.65-7.44) | 32.15 (25.53-39.49) | -0.4 (-0.44--0.35) |
| Palestine | 1219.5 (978.37-1480.79) | 151.54 (122.79-182.16) | 2587.42 (2059.84-3200.61) | 129.61 (104.67-157.84) | -0.74 (-0.84--0.64) |
| Panama | 597.35 (482.85-725.3) | 42.96 (34.65-52) | 1588.75 (1286.01-1903.1) | 38.51 (31.06-46.21) | -0.4 (-0.42--0.37) |
| Papua New Guinea | 467.76 (364.29-581.81) | 31.19 (25.15-37.89) | 1097.13 (849.76-1386.16) | 29.57 (23.43-36.33) | -0.12 (-0.15--0.1) |
| Paraguay | 728.37 (596.33-874.3) | 35.83 (29.3-42.88) | 1814.54 (1481.94-2182.59) | 34.96 (28.58-42.13) | -0.08 (-0.11--0.05) |
| Peru | 17565.95 (14328.9-21229.49) | 160.57 (131.3-193.14) | 50392.91 (41707.31-60250.36) | 161.46 (133.61-193.13) | 0 (-0.15-0.15) |
| Philippines | 26145.2 (21646.79-31223.24) | 103.24 (86.12-122.47) | 68991.71 (56928.47-82038.92) | 100.99 (84.51-119.42) | -0.06 (-0.13-0) |
| Poland | 27649.72 (22801.75-33006.84) | 64.28 (53.41-76.34) | 45822.08 (38020.98-54059.91) | 61.93 (51.51-73.13) | -0.19 (-0.23--0.14) |
| Portugal | 13301.97 (10976.78-15807.19) | 99.49 (83-117.62) | 22833.14 (19015.07-26896.11) | 82.38 (68.73-96.69) | -0.65 (-0.69--0.61) |
| Puerto Rico | 726.8 (573.27-895.55) | 19.92 (15.78-24.5) | 1409.72 (1113.67-1754.94) | 18.13 (14.24-22.58) | -0.31 (-0.35--0.27) |
| Qatar | 114.8 (92.31-141.1) | 168.12 (137.2-201.09) | 841.14 (653.64-1064.08) | 126.12 (103.08-155.48) | -1.02 (-1.14--0.9) |
| Republic of Korea | 6672.16 (5255.39-8382.57) | 30.3 (24.13-37.4) | 18766.86 (15099.49-22618.74) | 21.57 (17.44-25.97) | -1.17 (-1.3--1.03) |
| Republic of Moldova | 1428.99 (1114.73-1770.42) | 35.12 (27.74-42.97) | 2094 (1705-2546.36) | 35.42 (28.98-43.18) | 0.13 (0.02-0.23) |
| Romania | 18582.85 (14911.03-22452.69) | 69.1 (55.78-82.74) | 25576.31 (20455.13-30814.66) | 64.56 (51.94-77.49) | -0.26 (-0.29--0.23) |
| Russian Federation | 49092.21 (40119.08-58631.37) | 28.79 (23.7-34.22) | 63852.63 (52544.02-75881.11) | 26.42 (21.71-31.38) | -0.39 (-0.44--0.34) |
| Rwanda | 2981.78 (2312.65-3727.02) | 121.57 (96.49-150.04) | 4728.96 (3689.01-5871.36) | 94.61 (73.81-116.94) | -0.93 (-0.99--0.87) |
| Saint Kitts and Nevis | 8.22 (6.39-10.22) | 20.86 (16.57-25.48) | 11 (8.67-13.63) | 19.12 (15.09-23.47) | -0.25 (-0.28--0.22) |
| Saint Lucia | 18.22 (14.37-22.5) | 21.3 (16.91-25.92) | 41.22 (32.82-50.22) | 19.72 (15.63-24.05) | -0.21 (-0.24--0.17) |
| Saint Vincent and the Grenadines | 15.17 (12.07-18.75) | 21.29 (17.12-26.08) | 26.05 (20.7-32.33) | 19.84 (15.84-24.53) | -0.19 (-0.23--0.15) |
| Samoa | 31.76 (25.02-39.39) | 40.11 (31.77-49.13) | 46.27 (36.53-56.48) | 35.15 (28.18-42.76) | -0.37 (-0.42--0.32) |
| San Marino | 28.73 (23.88-34.17) | 86.41 (71.85-102.41) | 57.88 (48.61-68.49) | 77.97 (64.75-91.96) | -0.37 (-0.39--0.35) |
| Sao Tome and Principe | 114.06 (90.7-140.26) | 181.03 (146.53-219.82) | 163.62 (130.09-203.59) | 178.94 (143.5-220.92) | -0.03 (-0.04--0.03) |
| Saudi Arabia | 14122.85 (11390.54-17131.1) | 292.04 (234.93-355.35) | 29620.89 (23833.22-36081.02) | 232.31 (191.09-278.86) | -0.77 (-0.81--0.73) |
| Senegal | 4722.89 (3739.02-5824.79) | 162.07 (129.51-197.43) | 12835.51 (10294.97-15827.71) | 190.04 (154.05-232.91) | 0.66 (0.54-0.77) |
| Serbia | 7204.61 (5817.02-8727.18) | 67.58 (54.96-80.86) | 10593.57 (8497.17-13054.47) | 63.38 (51.28-77.3) | -0.27 (-0.3--0.24) |
| Seychelles | 59.91 (48.46-72.33) | 105.32 (85.18-126.98) | 94.67 (77.09-114.89) | 92.92 (75.85-112.72) | -0.43 (-0.44--0.43) |
| Sierra Leone | 1900.35 (1536.74-2314.15) | 108.67 (88.46-131.53) | 3251.53 (2647.89-3976.04) | 108.32 (88.75-130.89) | 0.06 (0.01-0.1) |
| Singapore | 486.85 (389.47-597.84) | 27.98 (22.58-34.02) | 1933.51 (1559.72-2342.29) | 26.37 (21.24-31.93) | -0.13 (-0.17--0.09) |
| Slovakia | 3959.15 (3196.72-4823.73) | 66.52 (54.04-80.68) | 5967.55 (4794.98-7314.23) | 62.84 (50.7-76.75) | -0.21 (-0.23--0.18) |
| Slovenia | 1564.74 (1255.01-1897.29) | 64.19 (51.65-77.39) | 2825.99 (2261.5-3461.86) | 60.96 (48.6-74.47) | -0.21 (-0.25--0.18) |
| Solomon Islands | 42.26 (32.74-53.31) | 39.17 (31.2-48.25) | 85.15 (66.84-106.38) | 36.2 (28.97-44.37) | -0.2 (-0.22--0.18) |
| Somalia | 1591.99 (1281.64-1964.34) | 83.35 (67.89-102.69) | 4320.2 (3433.76-5351.78) | 82.43 (66.33-101.19) | 0.2 (0.12-0.27) |
| South Africa | 8326.03 (6846.26-9932.7) | 45.01 (37.29-53.69) | 19438.86 (15920.22-23242.43) | 49.04 (40.27-58.31) | 0.39 (0.3-0.47) |
| South Sudan | 1360.16 (1105.7-1683.15) | 67.07 (54.77-81.62) | 1901.27 (1547.24-2319.97) | 62.57 (50.88-75.91) | -0.11 (-0.16--0.06) |
| Spain | 93874.06 (78058.95-110981.22) | 170.61 (143.36-201.53) | 166107.32 (139179.29-196140.58) | 150.56 (125.64-176.62) | -0.45 (-0.5--0.4) |
| Sri Lanka | 10288.31 (8291.17-12471.04) | 106.75 (87.09-129.07) | 23635.78 (19042.52-29106.15) | 93.05 (75.34-112.94) | -0.32 (-0.42--0.22) |
| Sudan | 13515.51 (10728.71-16685.93) | 162.47 (129.6-199.98) | 21757.34 (17852.06-25967.07) | 139.56 (114.6-166.44) | -0.4 (-0.45--0.34) |
| Suriname | 102.31 (82.23-124.61) | 42.39 (34.03-51.65) | 235.44 (193.36-284.67) | 41.08 (33.84-49.61) | 0.01 (-0.04-0.06) |
| Sweden | 12797.56 (10520.49-15428.5) | 77.02 (64.15-91.9) | 16806.57 (13961.16-20110.42) | 68.89 (57.29-82.16) | -0.49 (-0.53--0.46) |
| Switzerland | 9606.85 (7989.15-11391.92) | 86.01 (71.91-101.33) | 15427.27 (12845.06-18167.75) | 77.53 (64.41-91.16) | -0.37 (-0.39--0.35) |
| Syrian Arab Republic | 8752.35 (7069.21-10514.06) | 190.01 (155.37-229.03) | 18304.69 (14680.32-22204.33) | 163.52 (132.05-196.93) | -0.49 (-0.52--0.46) |
| Taiwan (Province of China) | 4244.44 (3374.84-5235.4) | 31.32 (25.33-38.38) | 14448.25 (11160.48-18046.54) | 35.77 (27.55-44.7) | 0.47 (0.28-0.66) |
| Tajikistan | 1954.62 (1589.13-2368.07) | 74.12 (60.17-89.86) | 2895.63 (2287.7-3646.65) | 73.51 (59-89.33) | -0.1 (-0.13--0.07) |
| Thailand | 41031.04 (34887.49-47253.92) | 128.77 (110.06-148.06) | 89062.99 (72422-106534.51) | 86.63 (70.55-103.35) | -1.74 (-1.96--1.51) |
| Timor-Leste | 309.61 (243.01-380.06) | 152.07 (121.63-186.13) | 893.36 (717.88-1088.9) | 119.72 (96.55-144.26) | -0.86 (-0.92--0.79) |
| Togo | 1575.25 (1268.23-1947) | 151.98 (123.56-184.9) | 5767.21 (4610.68-7162.25) | 186.32 (150.82-227.55) | 0.63 (0.54-0.72) |
| Tokelau | 0.57 (0.45-0.7) | 41.32 (32.61-50.53) | 0.44 (0.35-0.54) | 33.99 (27.28-41.54) | -0.61 (-0.64--0.59) |
| Tonga | 19.38 (15.4-23.7) | 38.82 (31.18-47.05) | 24.25 (19.41-29.57) | 31.97 (25.61-39.05) | -0.7 (-0.77--0.64) |
| Trinidad and Tobago | 163.75 (130.85-200.03) | 20.76 (16.68-25.22) | 353.82 (284.22-429.55) | 19.32 (15.6-23.42) | -0.24 (-0.26--0.22) |
| Tunisia | 8023.89 (6458.67-9754.57) | 171.44 (138.48-206.21) | 16695.61 (13540.87-20075.47) | 137.61 (112.17-165.2) | -0.76 (-0.8--0.73) |
| Turkey | 37347.58 (30227.21-45135.75) | 116.47 (94.69-140.82) | 91509.33 (73591.66-110807.14) | 107.43 (86.52-129.49) | -0.23 (-0.37--0.09) |
| Turkmenistan | 975.34 (766.28-1204.33) | 56.16 (44.55-69.09) | 1807.32 (1427.89-2270.56) | 52.17 (41.72-64.73) | -0.34 (-0.41--0.28) |
| Tuvalu | 2.63 (2.05-3.3) | 42.94 (34.22-53.07) | 3.46 (2.72-4.28) | 36.13 (28.72-44.19) | -0.52 (-0.56--0.48) |
| Uganda | 4820.27 (4140.85-5590.37) | 84.42 (72.65-97.03) | 10152.77 (8282.51-12319.53) | 86.85 (71-104.25) | 0.47 (0.14-0.8) |
| Ukraine | 18364.99 (15018.52-21980.4) | 26.21 (21.59-31.27) | 19836.09 (16335.39-23589.34) | 24.87 (20.56-29.5) | -0.26 (-0.28--0.23) |
| United Arab Emirates | 446.94 (359.94-548.34) | 180.6 (147.16-217.13) | 3196.06 (2461.16-4010.3) | 154.93 (125.9-186.36) | -0.44 (-0.47--0.41) |
| United Kingdom | 93090.33 (79518.6-108052.87) | 97.89 (83.96-112.76) | 127696.75 (109442.67-147639.28) | 91.69 (78.63-105.74) | -0.26 (-0.27--0.25) |
| United Republic of Tanzania | 18509.46 (15728.31-21801.91) | 192.28 (164.93-222.98) | 34628.06 (28513.02-41542.84) | 163.57 (135.03-195.5) | -0.65 (-0.83--0.46) |
| United States of America | 97961.97 (81821.6-115271.06) | 28.81 (24.12-33.8) | 167826.83 (140408.04-196426.48) | 27.37 (22.82-32.12) | -0.3 (-0.47--0.13) |
| United States Virgin Islands | 15.38 (12.28-19.03) | 20.76 (16.64-25.5) | 36.62 (28.89-46.1) | 19.14 (15.26-23.85) | -0.26 (-0.29--0.22) |
| Uruguay | 1709.35 (1374.89-2070.99) | 43.27 (35.02-52.2) | 2432.63 (1959.97-2941.55) | 39.93 (32.17-48.34) | -0.18 (-0.22--0.14) |
| Uzbekistan | 7683.22 (6181.42-9523.21) | 74.07 (59.59-90.72) | 11765.71 (9093.24-14781.56) | 72.77 (58.26-88.27) | -0.1 (-0.15--0.06) |
| Vanuatu | 22.73 (19.2-26.47) | 40.55 (34.47-47.11) | 50.21 (40.04-61.68) | 32.92 (26.52-40.07) | -0.75 (-0.89--0.61) |
| Venezuela (Bolivarian Republic of) | 4332.16 (3444.33-5323.09) | 51.32 (40.66-62.75) | 13278.4 (10517.59-16468.62) | 48.54 (38.64-60.12) | -0.09 (-0.13--0.06) |
| Viet Nam | 45777.99 (37651.73-54743.81) | 121.33 (100.33-144.05) | 87315.05 (73717.9-102954.25) | 103.89 (87.89-122.01) | -1.06 (-1.24--0.88) |
| Yemen | 6307.99 (5137.89-7677.63) | 151 (123.82-182.79) | 23382.14 (18939.02-28172.53) | 207.38 (169.36-249.24) | 0.99 (0.69-1.28) |
| Zambia | 1775.17 (1415.63-2180.08) | 74.53 (59.92-90.65) | 3245.56 (2568.28-3993.78) | 59.43 (47.37-73.4) | -0.78 (-0.87--0.69) |
| Zimbabwe | 1013.65 (797.45-1262.24) | 30.2 (24.15-36.98) | 1694.26 (1323.47-2094.45) | 30.18 (24.12-37) | 0.03 (-0.02-0.09) |

AMD, age-related macular degeneration; UI, uncertainty interval; CI, confidence interval; ASR, age-standardized rate; EAPC, estimated annual percentage change.

**Table S2. Estimated number and age-standardized rate (per 100,000 persons) of YLDs and temporal trends for AMD in 204 countries and territories from 1990 to 2019.**

| **Country** | **1990** | | **2019** | | **1990-2019** |
| --- | --- | --- | --- | --- | --- |
|  | **Number (95% UI)** | **ASR (95% UI)** | **Number (95% UI)** | **ASR (95% UI)** | **EAPC (95% CI)** |
| Afghanistan | 1064.5 (715.25-1515.32) | 15.98 (10.9-22.81) | 1629.18 (1091.83-2341.82) | 17.46 (11.62-25.01) | 0.55 (0.45-0.65) |
| Albania | 97.73 (65.65-139.1) | 5.29 (3.56-7.45) | 193.62 (128.27-277.78) | 4.39 (2.94-6.33) | -0.61 (-0.65--0.57) |
| Algeria | 1908.4 (1231.2-2801.83) | 16.76 (11.02-24.51) | 4062.02 (2694.73-5887.72) | 13.23 (8.86-19.03) | -0.76 (-0.78--0.74) |
| American Samoa | 0.81 (0.52-1.22) | 4.14 (2.7-6.14) | 1.56 (0.98-2.33) | 3.48 (2.24-5.14) | -0.51 (-0.57--0.46) |
| Andorra | 3.88 (2.59-5.64) | 8.54 (5.81-12.02) | 10.98 (7.41-15.41) | 7.31 (4.95-10.25) | -0.52 (-0.58--0.46) |
| Angola | 97.12 (62.53-144.57) | 3.33 (2.21-4.87) | 238.78 (149.85-354.32) | 2.84 (1.85-4.08) | -0.57 (-0.61--0.52) |
| Antigua and Barbuda | 0.91 (0.6-1.32) | 1.63 (1.07-2.39) | 1.31 (0.85-1.88) | 1.38 (0.9-1.98) | -0.56 (-0.59--0.52) |
| Argentina | 1073.63 (702.05-1581.71) | 3.57 (2.38-5.15) | 1620.85 (1088.23-2353.49) | 2.91 (1.95-4.22) | -0.64 (-0.66--0.61) |
| Armenia | 116.24 (77-168.63) | 4.79 (3.24-6.78) | 184.83 (124.26-263.02) | 4.4 (2.98-6.25) | -0.38 (-0.46--0.3) |
| Australia | 849.49 (566.97-1231.71) | 4.52 (3.04-6.54) | 1721.85 (1161.65-2482.86) | 3.79 (2.55-5.46) | -0.59 (-0.65--0.53) |
| Austria | 1168.43 (786.86-1665.71) | 9.51 (6.4-13.41) | 1540.65 (1046.26-2161.77) | 7.62 (5.15-10.7) | -0.75 (-0.78--0.72) |
| Azerbaijan | 229.9 (154.54-331.09) | 5.11 (3.44-7.25) | 366.04 (243.71-522.79) | 4.6 (3.1-6.54) | -0.45 (-0.57--0.34) |
| Bahamas | 2.28 (1.46-3.39) | 1.67 (1.08-2.46) | 5.12 (3.29-7.23) | 1.44 (0.93-2.05) | -0.47 (-0.53--0.4) |
| Bahrain | 21.5 (14.09-31.47) | 15.77 (10.28-22.82) | 86.86 (57.54-124.94) | 11.53 (7.68-16.54) | -1.11 (-1.15--1.07) |
| Bangladesh | 3984.2 (2734.28-5612.92) | 9.67 (6.7-13.51) | 9511.81 (6531.29-13547.71) | 7.69 (5.3-10.87) | -0.7 (-0.81--0.59) |
| Barbados | 1.77 (1.15-2.6) | 0.57 (0.37-0.83) | 2.58 (1.7-3.72) | 0.51 (0.34-0.73) | -0.27 (-0.31--0.23) |
| Belarus | 279.01 (179.59-405.86) | 2.17 (1.42-3.12) | 304.22 (199.74-445.35) | 1.85 (1.21-2.68) | -0.65 (-0.71--0.59) |
| Belgium | 1484.48 (994.79-2131.25) | 9.39 (6.29-13.46) | 1985.7 (1379.33-2793.65) | 7.58 (5.26-10.67) | -0.74 (-0.77--0.71) |
| Belize | 1.56 (1.01-2.29) | 1.76 (1.14-2.57) | 3.62 (2.32-5.21) | 1.48 (0.96-2.15) | -0.57 (-0.62--0.52) |
| Benin | 121.56 (82.33-172.81) | 6.82 (4.64-9.59) | 460.68 (314.51-655.1) | 11.04 (7.55-15.68) | 1.52 (1.35-1.69) |
| Bermuda | 0.93 (0.59-1.38) | 1.55 (1.01-2.3) | 1.72 (1.11-2.49) | 1.26 (0.81-1.82) | -0.69 (-0.74--0.64) |
| Bhutan | 10.31 (7.04-14.91) | 5.32 (3.69-7.62) | 19.02 (12.81-27.04) | 3.72 (2.52-5.25) | -1.32 (-1.5--1.13) |
| Bolivia (Plurinational State of) | 190.54 (127.32-277.84) | 6.78 (4.58-9.85) | 456.56 (304.35-658.98) | 5.57 (3.75-7.97) | -0.64 (-0.66--0.61) |
| Bosnia and Herzegovina | 182.72 (123.79-268.31) | 5.16 (3.55-7.48) | 258.37 (170.57-370.84) | 4.28 (2.83-6.14) | -0.77 (-0.85--0.68) |
| Botswana | 16.37 (9.98-25.37) | 3.45 (2.15-5.25) | 27.31 (16.66-41.83) | 2.52 (1.58-3.75) | -1.12 (-1.2--1.04) |
| Brazil | 4074.84 (2837.34-5602.63) | 5.17 (3.59-7.12) | 11186.9 (7731.88-15416.76) | 4.87 (3.37-6.74) | -0.09 (-0.25-0.07) |
| Brunei Darussalam | 1.42 (0.89-2.15) | 2.38 (1.55-3.53) | 4.29 (2.76-6.5) | 2.23 (1.49-3.28) | -0.21 (-0.29--0.12) |
| Bulgaria | 476.89 (331.74-660.6) | 4.03 (2.82-5.48) | 563.22 (377.87-793.4) | 3.65 (2.46-5.16) | -0.26 (-0.32--0.19) |
| Burkina Faso | 162.3 (106.02-238.17) | 4.51 (2.96-6.46) | 646.28 (424.61-926.77) | 8.29 (5.56-11.94) | 1.48 (1.11-1.85) |
| Burundi | 152.37 (98.6-221.36) | 7.49 (4.92-10.84) | 216.03 (142.4-314.56) | 5.9 (3.94-8.49) | -0.84 (-0.93--0.76) |
| Cabo Verde | 29.92 (20.56-41.63) | 12.71 (8.74-17.69) | 41.53 (28.27-58.6) | 10.45 (7.1-14.68) | -0.71 (-0.76--0.66) |
| Cambodia | 300.89 (196.46-441.26) | 7.77 (5.08-11.17) | 634.27 (414.19-921.18) | 5.95 (3.91-8.68) | -0.85 (-0.97--0.74) |
| Cameroon | 396.25 (269.49-557.04) | 10.67 (7.3-14.88) | 1032.77 (708.89-1474.28) | 10.53 (7.24-14.96) | -0.19 (-0.3--0.08) |
| Canada | 874.45 (583.6-1270.58) | 2.73 (1.82-3.95) | 1895.3 (1251.61-2723.26) | 2.52 (1.65-3.62) | -0.29 (-0.3--0.28) |
| Central African Republic | 12.01 (7.79-17.76) | 1.62 (1.09-2.35) | 24.4 (15.63-35.71) | 1.82 (1.19-2.68) | 0.5 (0.46-0.53) |
| Chad | 202.95 (135.41-294.97) | 7.79 (5.23-11.32) | 502.89 (340.98-718.55) | 10.58 (7.26-15.12) | 1.17 (1.02-1.32) |
| Chile | 244.96 (160-363.49) | 2.78 (1.83-4.05) | 559.29 (368.53-820.33) | 2.32 (1.53-3.4) | -0.66 (-0.69--0.64) |
| China | 54885.3 (37530.29-77765.66) | 7.49 (5.16-10.54) | 142065.76 (97100.02-197277.11) | 7.22 (4.91-10.06) | -0.53 (-0.81--0.26) |
| Colombia | 718.05 (480.43-1038.93) | 4.71 (3.17-6.7) | 2033.16 (1375.82-2881.98) | 3.8 (2.57-5.39) | -0.67 (-0.72--0.61) |
| Comoros | 27.8 (17.84-41.3) | 13.73 (9.04-19.92) | 47.34 (31.42-67.68) | 10.64 (7.08-15.2) | -0.91 (-0.94--0.88) |
| Congo | 26.42 (17.4-38.71) | 3.06 (2.04-4.39) | 53.72 (34.28-78.33) | 2.68 (1.75-3.84) | -0.54 (-0.59--0.5) |
| Cook Islands | 0.81 (0.5-1.24) | 6.89 (4.4-10.35) | 1.25 (0.78-1.87) | 4.97 (3.12-7.45) | -0.84 (-0.95--0.73) |
| Costa Rica | 63.56 (42.96-91.21) | 3.91 (2.64-5.59) | 177.37 (120.34-253.7) | 3.49 (2.36-5) | -0.44 (-0.47--0.4) |
| Côte d'Ivoire | 134.06 (88.42-191.65) | 4.89 (3.29-6.92) | 1031 (697.31-1458.12) | 11.83 (8.12-16.56) | 1.48 (0.8-2.17) |
| Croatia | 271.88 (179.86-385.92) | 4.41 (2.96-6.24) | 364.61 (243.65-519.41) | 3.92 (2.61-5.55) | -0.43 (-0.47--0.4) |
| Cuba | 219.34 (145.99-314.58) | 2.16 (1.44-3.07) | 368.04 (241.07-535.92) | 1.87 (1.22-2.72) | -0.38 (-0.49--0.28) |
| Cyprus | 62.23 (40.82-90.8) | 8.81 (5.93-12.64) | 133.07 (90.33-191.37) | 7.18 (4.88-10.15) | -0.72 (-0.76--0.67) |
| Czechia | 612.48 (407.16-855.9) | 4.44 (2.98-6.2) | 851.29 (575.63-1189.37) | 3.84 (2.6-5.37) | -0.43 (-0.48--0.38) |
| Democratic People's Republic of Korea | 323.44 (211.05-461.27) | 2.56 (1.68-3.61) | 651.38 (408.42-934.54) | 2.17 (1.37-3.1) | -0.63 (-0.68--0.58) |
| Democratic Republic of the Congo | 172.43 (109.83-249.56) | 1.47 (0.99-2.13) | 430.74 (282.12-624.16) | 1.66 (1.09-2.39) | 0.58 (0.47-0.7) |
| Denmark | 476.94 (327.74-671.59) | 5.54 (3.78-7.76) | 599.4 (409.87-868.85) | 4.74 (3.22-6.87) | -0.5 (-0.55--0.46) |
| Djibouti | 14.18 (9.16-20.5) | 13.24 (8.74-19.28) | 48.19 (31.65-69.82) | 10.08 (6.69-14.59) | -1.02 (-1.1--0.95) |
| Dominica | 1.27 (0.81-1.89) | 1.68 (1.08-2.5) | 1.35 (0.86-1.97) | 1.47 (0.94-2.12) | -0.41 (-0.47--0.34) |
| Dominican Republic | 84.11 (54.15-127.13) | 2.52 (1.65-3.75) | 169.5 (108.37-251.89) | 1.93 (1.24-2.85) | -0.82 (-0.9--0.75) |
| Ecuador | 137.4 (90.84-199) | 2.84 (1.89-4.08) | 339.67 (225.76-494.16) | 2.35 (1.58-3.4) | -0.58 (-0.64--0.53) |
| Egypt | 4288.09 (2837.63-6168.46) | 16.85 (11.12-24.27) | 7140.57 (4686.19-10427.8) | 12.9 (8.48-18.52) | -0.84 (-0.86--0.82) |
| El Salvador | 160.18 (106.82-231.26) | 5.79 (3.85-8.35) | 261.7 (176.19-380.2) | 4.32 (2.9-6.25) | -0.87 (-0.97--0.78) |
| Equatorial Guinea | 5.65 (3.61-8.59) | 3.51 (2.3-5.19) | 11 (7.07-16.5) | 2.89 (1.88-4.26) | -0.77 (-0.85--0.68) |
| Eritrea | 83.02 (52.49-122.66) | 11.54 (7.42-17.1) | 197.48 (126.22-294.94) | 9.62 (6.12-14.01) | -0.75 (-0.89--0.62) |
| Estonia | 29.65 (19.17-43.57) | 1.45 (0.95-2.14) | 35.11 (23.49-50.41) | 1.21 (0.8-1.74) | -0.84 (-0.89--0.78) |
| Eswatini | 7.67 (4.99-11.42) | 3.22 (2.11-4.76) | 14.02 (8.9-20.76) | 2.96 (1.96-4.37) | -0.28 (-0.36--0.19) |
| Ethiopia | 2015.29 (1319.82-2912.41) | 12.15 (8.14-17.26) | 4711.97 (3111.14-6725.97) | 13.11 (8.64-18.85) | 0.41 (0.28-0.54) |
| Fiji | 15.93 (10.09-24) | 5.37 (3.5-7.97) | 32.24 (20.87-47.63) | 4.91 (3.23-7.14) | -0.12 (-0.2--0.04) |
| Finland | 682.35 (458.55-980.38) | 9.61 (6.44-13.71) | 1042.4 (712.97-1495.21) | 7.49 (5.08-10.67) | -0.92 (-0.99--0.86) |
| France | 6212.6 (4242-8914.62) | 7.03 (4.82-10.02) | 8943.44 (6178.14-12549.56) | 5.51 (3.78-7.72) | -0.86 (-0.9--0.82) |
| Gabon | 14.77 (9.7-21.72) | 3.01 (2.02-4.35) | 21.74 (14.04-32) | 2.52 (1.65-3.66) | -0.59 (-0.63--0.54) |
| Gambia | 20.12 (13.46-28.19) | 6.65 (4.47-9.2) | 80.84 (54.98-115.28) | 9.52 (6.5-13.5) | 1.46 (1.29-1.64) |
| Georgia | 279.3 (186.84-402.27) | 4.74 (3.22-6.77) | 291.7 (200.42-408.12) | 4.58 (3.13-6.47) | -0.09 (-0.15--0.02) |
| Germany | 12187.47 (8223.33-17344.71) | 9.28 (6.27-13.16) | 15951.55 (11045.36-22474.88) | 7.3 (5.01-10.33) | -0.76 (-0.82--0.71) |
| Ghana | 445.18 (298.75-637.48) | 8.81 (5.97-12.49) | 1593.33 (1062.66-2229.31) | 11.47 (7.69-16.07) | 0.64 (0.41-0.88) |
| Greece | 1303.73 (864.24-1879.12) | 8.72 (5.89-12.46) | 2009.52 (1364.87-2894.15) | 7.17 (4.84-10.29) | -0.66 (-0.73--0.59) |
| Greenland | 0.84 (0.54-1.23) | 3.51 (2.32-5.17) | 1.89 (1.26-2.72) | 3.26 (2.18-4.72) | -0.17 (-0.2--0.13) |
| Grenada | 1.36 (0.9-1.97) | 1.75 (1.14-2.52) | 1.47 (0.93-2.13) | 1.42 (0.91-2.06) | -0.61 (-0.69--0.54) |
| Guam | 2.53 (1.61-3.83) | 3.85 (2.55-5.76) | 6.29 (4.05-9.23) | 3.33 (2.14-4.89) | -0.3 (-0.38--0.21) |
| Guatemala | 172.07 (111.77-259.49) | 5.65 (3.74-8.28) | 455.45 (296.57-668.72) | 4.41 (2.88-6.39) | -0.78 (-0.9--0.66) |
| Guinea | 274.58 (183.66-393.02) | 8.96 (6.06-12.85) | 601.89 (407.97-853.53) | 12.14 (8.24-17.17) | 0.91 (0.75-1.06) |
| Guinea-Bissau | 36.52 (24.57-52.5) | 10.66 (7.26-15.17) | 91.72 (62.04-128.89) | 15.49 (10.64-21.79) | 1.02 (0.78-1.26) |
| Guyana | 5.94 (3.83-8.72) | 1.82 (1.19-2.67) | 8.35 (5.33-12.19) | 1.53 (0.98-2.23) | -0.47 (-0.54--0.4) |
| Haiti | 58.92 (38.42-88.32) | 2.16 (1.42-3.16) | 107.26 (68.27-160.79) | 1.83 (1.19-2.69) | -0.54 (-0.57--0.51) |
| Honduras | 78.69 (51.8-113.3) | 4.37 (2.87-6.24) | 190.8 (126.82-275.56) | 3.57 (2.39-5.12) | -0.7 (-0.75--0.65) |
| Hungary | 839.41 (559.13-1212.55) | 5.69 (3.84-8.14) | 1007.63 (667.67-1406.68) | 4.95 (3.31-6.94) | -0.42 (-0.49--0.36) |
| Iceland | 66.3 (45.5-92.02) | 22.51 (15.5-31.39) | 88.09 (59.49-126.52) | 14.43 (9.76-20.75) | -1.79 (-1.95--1.64) |
| India | 48642.74 (32473.63-68389.89) | 12.58 (8.57-17.65) | 86263.16 (59285.66-122397.81) | 8.16 (5.67-11.52) | -2 (-2.16--1.83) |
| Indonesia | 7568.39 (4862.89-11185.53) | 9.19 (6.01-13.33) | 15549.29 (10034.95-22824.29) | 8.3 (5.38-12.03) | -0.4 (-0.46--0.33) |
| Iran (Islamic Republic of) | 5959.63 (4013.67-8466.99) | 27.03 (18.17-38.21) | 14120.3 (9634.01-19990.03) | 21.6 (14.74-30.5) | -0.68 (-0.78--0.57) |
| Iraq | 1141.66 (746.76-1643.39) | 16.37 (10.78-23.4) | 2579.32 (1720.99-3711.01) | 13.35 (8.9-19.23) | -0.75 (-0.82--0.68) |
| Ireland | 371.53 (250.69-532) | 9.4 (6.29-13.42) | 565.12 (382.73-813.15) | 7.29 (4.92-10.5) | -0.91 (-0.94--0.87) |
| Israel | 444.51 (293.44-641.49) | 9.55 (6.46-13.6) | 945.5 (633.46-1325.42) | 7.63 (5.13-10.81) | -0.78 (-0.85--0.7) |
| Italy | 18923.01 (12947.73-27163.05) | 21.11 (14.23-30.1) | 25590.06 (17489.76-36523.36) | 15.16 (10.37-21.52) | -1.21 (-1.33--1.09) |
| Jamaica | 29.88 (19.46-43.25) | 1.64 (1.06-2.37) | 41.23 (27.29-59.02) | 1.36 (0.89-1.94) | -0.63 (-0.7--0.56) |
| Japan | 3729.63 (2480.02-5306.73) | 2.31 (1.53-3.29) | 8616.81 (5823.69-12286.8) | 2 (1.34-2.84) | -0.55 (-0.69--0.41) |
| Jordan | 187.42 (118.79-278.3) | 17.69 (11.29-26.68) | 645.36 (423.23-936.65) | 12.14 (7.96-17.57) | -1.41 (-1.46--1.35) |
| Kazakhstan | 572.42 (384.46-816.75) | 4.99 (3.36-7.06) | 710.87 (477.39-1016.71) | 4.56 (3.08-6.46) | -0.4 (-0.53--0.26) |
| Kenya | 2512.19 (1606.01-3691.81) | 34.75 (22.63-50.85) | 3216.9 (2078.16-4679.22) | 17.43 (11.28-25.1) | -1.47 (-1.84--1.1) |
| Kiribati | 1.48 (0.94-2.25) | 4.76 (3.12-7) | 2.28 (1.4-3.46) | 4.01 (2.56-5.94) | -0.52 (-0.56--0.48) |
| Kuwait | 62.96 (41.53-90.13) | 13.95 (9.24-19.83) | 221.13 (148.43-313.62) | 11.25 (7.54-15.96) | -0.72 (-0.74--0.7) |
| Kyrgyzstan | 217.78 (147.7-303.52) | 7.5 (5.09-10.45) | 296.42 (197.58-416.84) | 7.43 (5.03-10.53) | 0.02 (-0.05-0.1) |
| Lao People's Democratic Republic | 175.61 (116.54-252.32) | 9.98 (6.73-14.17) | 345.21 (233.57-490.66) | 9.37 (6.35-13.2) | -0.33 (-0.42--0.24) |
| Latvia | 77.84 (50.98-113.68) | 2.16 (1.42-3.14) | 81.42 (53.31-119.66) | 1.87 (1.22-2.73) | -0.65 (-0.7--0.61) |
| Lebanon | 317.79 (218.44-444.87) | 14.79 (10.25-20.51) | 642.52 (425.47-907.57) | 12.41 (8.22-17.43) | -0.32 (-0.47--0.16) |
| Lesotho | 28.91 (18.63-42.56) | 3.33 (2.16-4.86) | 33.2 (21.66-48.45) | 3.12 (2.04-4.55) | -0.17 (-0.24--0.11) |
| Liberia | 114.03 (76.88-164.34) | 10.89 (7.35-15.65) | 169.67 (116.82-236.69) | 10.19 (7.01-14.3) | -0.27 (-0.31--0.23) |
| Libya | 199.9 (132.55-290.38) | 12.12 (8.02-17.59) | 384.77 (263.92-546.05) | 8.87 (6.1-12.6) | -1.03 (-1.15--0.91) |
| Lithuania | 95.64 (62.07-138.83) | 2.1 (1.38-3.04) | 118.96 (77.54-176.33) | 1.9 (1.24-2.8) | -0.45 (-0.49--0.41) |
| Luxembourg | 50.15 (33.57-70.6) | 9.25 (6.23-13.07) | 78.9 (53.41-112.18) | 7.25 (4.93-10.31) | -0.83 (-0.89--0.78) |
| Madagascar | 688.25 (456.82-984.18) | 15.26 (10.24-21.86) | 1022.84 (680.56-1465.98) | 11.87 (7.97-17.01) | -0.88 (-0.93--0.83) |
| Malawi | 391.05 (250.4-574.93) | 11.65 (7.61-16.85) | 540.31 (355.05-771.51) | 8.64 (5.8-12.24) | -0.8 (-0.94--0.67) |
| Malaysia | 1717.48 (1134.78-2441) | 21.06 (13.88-29.94) | 2843.39 (1901.63-4129.07) | 11.35 (7.72-16.32) | -2.62 (-2.87--2.37) |
| Maldives | 6.18 (3.99-9.03) | 8.76 (5.76-12.71) | 15.53 (10.18-22.27) | 6.21 (4.1-8.96) | -1.21 (-1.3--1.12) |
| Mali | 417.56 (286.54-595.39) | 11.37 (7.91-16.21) | 724.49 (471.23-1050.55) | 9.51 (6.22-13.7) | -0.83 (-1.05--0.6) |
| Malta | 36.83 (24.77-52.68) | 9.29 (6.33-13.23) | 77.45 (52.28-110.55) | 7.66 (5.12-10.86) | -0.65 (-0.68--0.62) |
| Marshall Islands | 0.65 (0.41-0.98) | 4.63 (3.02-6.82) | 1.12 (0.7-1.69) | 3.83 (2.52-5.57) | -0.64 (-0.67--0.6) |
| Mauritania | 114.85 (77.12-162.29) | 12.39 (8.35-17.49) | 213.49 (142.98-298.49) | 11.17 (7.56-15.55) | -0.32 (-0.34--0.31) |
| Mauritius | 55.21 (37.44-78.54) | 8.38 (5.69-11.91) | 110.87 (73.63-160.1) | 6.49 (4.36-9.29) | -0.91 (-0.93--0.89) |
| Mexico | 2203.4 (1514.3-3196.31) | 5.86 (3.98-8.41) | 5162.41 (3531.65-7376.06) | 4.69 (3.21-6.68) | -0.73 (-0.79--0.67) |
| Micronesia (Federated States of) | 1.95 (1.25-2.9) | 4.74 (3.13-7.07) | 2.25 (1.39-3.47) | 3.71 (2.35-5.49) | -0.76 (-0.8--0.72) |
| Monaco | 6.83 (4.67-9.71) | 8.52 (5.75-12.03) | 8.14 (5.5-11.35) | 7.33 (4.94-10.12) | -0.53 (-0.56--0.5) |
| Mongolia | 44.85 (29.28-66.44) | 4.88 (3.21-7.16) | 80.11 (51.73-119.62) | 4.42 (2.95-6.46) | -0.29 (-0.36--0.22) |
| Montenegro | 26.39 (17.65-37.47) | 4.48 (3.01-6.27) | 40.95 (27.4-58.28) | 4.13 (2.78-5.89) | -0.31 (-0.36--0.26) |
| Morocco | 1210.31 (831.67-1667.15) | 9.62 (6.73-13.15) | 2500.18 (1638.35-3577.89) | 8.81 (5.81-12.49) | -0.09 (-0.23-0.05) |
| Mozambique | 492.16 (314.19-730.18) | 9.69 (6.27-14.29) | 787.47 (507.89-1156) | 8.74 (5.64-12.73) | -0.21 (-0.31--0.11) |
| Myanmar | 1168.57 (752.4-1724.36) | 5.79 (3.79-8.46) | 2185.4 (1422.72-3272.69) | 5.22 (3.37-7.76) | -0.34 (-0.64--0.03) |
| Namibia | 21.24 (13.83-31.66) | 3.29 (2.2-4.82) | 34.31 (22.55-50.23) | 2.78 (1.83-4.05) | -0.61 (-0.67--0.55) |
| Nauru | 0.12 (0.07-0.18) | 4.11 (2.69-6.02) | 0.1 (0.06-0.16) | 3.44 (2.16-5.11) | -0.45 (-0.5--0.4) |
| Nepal | 1323.82 (888.58-1884.47) | 16.57 (11.14-23.33) | 4361.2 (2951.64-6178.19) | 20.71 (14.06-28.91) | 0.52 (0.38-0.67) |
| Netherlands | 1565.45 (1059.96-2209.07) | 7.66 (5.22-10.87) | 2255.34 (1552.6-3168.9) | 6.08 (4.17-8.5) | -0.86 (-0.89--0.82) |
| New Zealand | 206 (135.87-295.48) | 5.34 (3.51-7.62) | 358.92 (242.49-509.9) | 4.25 (2.84-6.07) | -0.79 (-0.83--0.75) |
| Nicaragua | 65.83 (43.75-94.75) | 4.95 (3.3-7.02) | 155.4 (103.81-224.14) | 3.97 (2.66-5.68) | -0.8 (-0.84--0.77) |
| Niger | 192.57 (127.69-275.71) | 8.43 (5.68-12.14) | 693.13 (464.48-997.77) | 10.37 (7.01-14.82) | 0.49 (0.35-0.63) |
| Nigeria | 8375.73 (5838.04-11564.16) | 20.85 (14.53-28.73) | 14332.25 (9884.59-19970.23) | 19.15 (13.27-26.73) | -0.61 (-0.8--0.42) |
| Niue | 0.09 (0.06-0.13) | 4.07 (2.63-6) | 0.07 (0.04-0.1) | 3.08 (2.02-4.54) | -0.89 (-0.95--0.84) |
| North Macedonia | 83.95 (56.32-118.75) | 4.97 (3.38-7) | 130.25 (87.32-184.49) | 4.22 (2.83-5.95) | -0.55 (-0.6--0.5) |
| Northern Mariana Islands | 0.46 (0.29-0.7) | 3.66 (2.4-5.43) | 1.47 (0.93-2.17) | 3.04 (1.99-4.39) | -0.43 (-0.53--0.34) |
| Norway | 580.24 (405.65-808.38) | 7.84 (5.44-10.92) | 736.09 (513.66-1010.53) | 6.92 (4.81-9.57) | -0.44 (-0.49--0.39) |
| Oman | 74.13 (47.84-107.47) | 13.67 (8.99-19.56) | 231.96 (153.41-328.09) | 17.27 (11.78-24.55) | 0.36 (-0.13-0.84) |
| Pakistan | 7557.08 (5267.01-10499.22) | 14.39 (10.07-20.05) | 10662.61 (7408.09-14764.82) | 11.3 (7.91-15.49) | -0.93 (-1.02--0.83) |
| Palau | 0.34 (0.22-0.5) | 3.84 (2.51-5.59) | 0.58 (0.37-0.87) | 3.04 (1.99-4.5) | -0.67 (-0.74--0.61) |
| Palestine | 96.33 (64.47-136.62) | 11.99 (8.01-16.97) | 190.23 (130.51-271.6) | 9.61 (6.64-13.63) | -1.3 (-1.53--1.07) |
| Panama | 60.11 (39.65-87.46) | 4.32 (2.86-6.25) | 151.62 (100.95-219.03) | 3.67 (2.44-5.31) | -0.51 (-0.55--0.46) |
| Papua New Guinea | 42.78 (27.25-64.16) | 2.77 (1.84-4.02) | 93.53 (60.8-138.43) | 2.44 (1.62-3.53) | -0.32 (-0.37--0.27) |
| Paraguay | 62.21 (41.3-87.56) | 3.04 (2.03-4.27) | 137.96 (92.52-197.94) | 2.64 (1.78-3.75) | -0.5 (-0.55--0.44) |
| Peru | 1381.48 (933.66-1954.91) | 12.64 (8.5-17.96) | 3595.59 (2462-4995.65) | 11.49 (7.87-15.96) | -0.34 (-0.45--0.23) |
| Philippines | 2142.43 (1462.91-3049.89) | 8.49 (5.83-11.98) | 5392.53 (3665.52-7632.45) | 7.96 (5.47-11.21) | -0.2 (-0.28--0.12) |
| Poland | 1903.21 (1308.47-2653.77) | 4.46 (3.08-6.19) | 3035.59 (2070.09-4248.08) | 4.12 (2.82-5.8) | -0.3 (-0.35--0.24) |
| Portugal | 1376.03 (928.72-1965.82) | 10.37 (7.07-14.69) | 2165.87 (1473.05-3090.79) | 7.86 (5.33-11.23) | -0.94 (-1--0.87) |
| Puerto Rico | 53.95 (34.81-79.82) | 1.49 (0.97-2.18) | 94.28 (60.84-135.86) | 1.21 (0.77-1.76) | -0.75 (-0.82--0.67) |
| Qatar | 9.61 (6.35-13.86) | 13.94 (9.35-20.06) | 52.62 (34.83-75.48) | 7.8 (5.49-10.95) | -2.17 (-2.46--1.88) |
| Republic of Korea | 769.37 (479.95-1126.17) | 3.4 (2.22-4.92) | 1815.85 (1193.33-2609.23) | 2.08 (1.38-2.99) | -1.73 (-1.91--1.55) |
| Republic of Moldova | 133.01 (86.1-194.91) | 3.26 (2.15-4.75) | 174.94 (116.8-246.09) | 2.97 (1.99-4.16) | -0.29 (-0.39--0.18) |
| Romania | 1358.25 (909.11-1962.98) | 5.09 (3.42-7.35) | 1730.66 (1154.7-2456.27) | 4.4 (2.97-6.27) | -0.48 (-0.52--0.45) |
| Russian Federation | 3991.9 (2649.28-5752.46) | 2.34 (1.59-3.37) | 4805.42 (3232.53-6812.15) | 1.99 (1.35-2.84) | -0.77 (-0.84--0.69) |
| Rwanda | 318.28 (203.2-468.4) | 12.83 (8.3-18.57) | 433.49 (282.1-636.75) | 8.58 (5.63-12.51) | -1.46 (-1.54--1.38) |
| Saint Kitts and Nevis | 0.63 (0.41-0.91) | 1.62 (1.07-2.33) | 0.78 (0.51-1.17) | 1.35 (0.88-1.97) | -0.56 (-0.61--0.52) |
| Saint Lucia | 1.42 (0.92-2.08) | 1.68 (1.09-2.42) | 2.97 (1.95-4.28) | 1.42 (0.93-2.03) | -0.52 (-0.57--0.46) |
| Saint Vincent and the Grenadines | 1.18 (0.74-1.76) | 1.67 (1.06-2.47) | 1.87 (1.2-2.79) | 1.43 (0.92-2.11) | -0.51 (-0.58--0.45) |
| Samoa | 3.48 (2.2-5.19) | 4.32 (2.77-6.36) | 4.65 (2.94-6.81) | 3.45 (2.25-5.03) | -0.64 (-0.71--0.57) |
| San Marino | 2.84 (1.93-4.07) | 8.61 (5.85-12.23) | 5.45 (3.69-7.6) | 7.34 (4.99-10.26) | -0.55 (-0.59--0.52) |
| Sao Tome and Principe | 7.71 (5.18-11.11) | 12.39 (8.35-17.59) | 10.12 (6.83-14.19) | 11.16 (7.65-15.69) | -0.35 (-0.36--0.34) |
| Saudi Arabia | 1443.22 (949.59-2049.02) | 29.6 (19.51-42.16) | 2677.34 (1754.9-3866.75) | 21.02 (13.88-30.23) | -1.24 (-1.31--1.18) |
| Senegal | 325.93 (222.37-461.72) | 11.28 (7.71-15.98) | 814.49 (546.37-1168.34) | 12.15 (8.21-17.24) | 0.34 (0.27-0.41) |
| Serbia | 521.58 (344.81-746.04) | 4.9 (3.3-6.96) | 689.98 (457.14-977.52) | 4.2 (2.81-5.94) | -0.55 (-0.59--0.5) |
| Seychelles | 5.24 (3.48-7.44) | 9.24 (6.15-13.1) | 7.41 (5-10.73) | 7.33 (4.96-10.47) | -0.8 (-0.8--0.79) |
| Sierra Leone | 134.22 (91.71-190.03) | 7.75 (5.33-10.96) | 216.75 (145.68-308.15) | 7.26 (4.96-10.29) | -0.09 (-0.17--0.02) |
| Singapore | 47.01 (30.26-68.6) | 2.67 (1.76-3.83) | 185.73 (121.01-270.22) | 2.51 (1.66-3.64) | -0.14 (-0.19--0.09) |
| Slovakia | 277.56 (186.27-397.72) | 4.71 (3.16-6.67) | 390.44 (260.42-551.53) | 4.14 (2.76-5.83) | -0.38 (-0.41--0.34) |
| Slovenia | 106.09 (72.19-149.79) | 4.37 (3-6.18) | 178.18 (119.02-250.18) | 3.84 (2.58-5.44) | -0.48 (-0.54--0.43) |
| Solomon Islands | 4.75 (2.95-7.19) | 4.16 (2.7-6.13) | 8.77 (5.43-13.16) | 3.55 (2.3-5.14) | -0.41 (-0.45--0.37) |
| Somalia | 185 (118.69-270.7) | 9.6 (6.24-14.12) | 486.44 (303.34-731.19) | 9.24 (5.99-13.65) | 0.21 (0.1-0.32) |
| South Africa | 576.74 (392.47-815.86) | 3.09 (2.11-4.34) | 1364.55 (929.92-1948.58) | 3.42 (2.35-4.86) | 0.51 (0.35-0.68) |
| South Sudan | 141.9 (91.25-206.75) | 7.01 (4.56-10.2) | 183.9 (122.48-268.1) | 6.09 (4.06-8.87) | -0.33 (-0.4--0.27) |
| Spain | 10517.1 (7093.87-15007.72) | 19.22 (13.08-27.12) | 17353.3 (11945.66-24367.71) | 15.82 (10.86-22.11) | -0.71 (-0.79--0.63) |
| Sri Lanka | 779.23 (519.47-1108.06) | 8.17 (5.46-11.54) | 1583.33 (1058.63-2275.35) | 6.33 (4.28-8.93) | -0.76 (-0.86--0.65) |
| Sudan | 1447.32 (915.69-2173.09) | 17.37 (11.02-25.65) | 2171.43 (1422.14-3095.28) | 13.94 (9.17-20.11) | -0.62 (-0.69--0.54) |
| Suriname | 8.02 (5.15-11.58) | 3.3 (2.14-4.73) | 17.66 (11.85-24.79) | 3.07 (2.07-4.29) | -0.13 (-0.21--0.04) |
| Sweden | 1462.42 (989.63-2115.03) | 8.84 (5.97-12.79) | 1811.11 (1249.6-2615.68) | 7.44 (5.11-10.71) | -0.66 (-0.69--0.63) |
| Switzerland | 941.57 (639.05-1326.22) | 8.5 (5.78-11.99) | 1445.46 (994.99-2037.86) | 7.27 (4.98-10.27) | -0.53 (-0.56--0.49) |
| Syrian Arab Republic | 798.39 (519.96-1155.91) | 17.25 (11.26-24.78) | 1510.14 (990.32-2159.9) | 13.47 (8.95-19.41) | -0.86 (-0.92--0.81) |
| Taiwan (Province of China) | 145.23 (89.34-212.54) | 1.08 (0.68-1.58) | 445.77 (262.87-673.08) | 1.1 (0.65-1.65) | 0.03 (-0.11-0.18) |
| Tajikistan | 136.9 (92.08-193.99) | 5.17 (3.5-7.29) | 192.38 (126.11-273.99) | 4.89 (3.29-6.95) | -0.24 (-0.31--0.16) |
| Thailand | 2438.17 (1682.47-3332.52) | 7.83 (5.42-10.76) | 4431.68 (2918.91-6291.74) | 4.33 (2.86-6.1) | -2.53 (-2.77--2.29) |
| Timor-Leste | 33.12 (21.54-48.11) | 15.99 (10.6-23.24) | 88.06 (58.44-126.29) | 11.82 (7.89-16.88) | -1.13 (-1.2--1.06) |
| Togo | 104.72 (70.54-149.52) | 10.25 (6.93-14.54) | 366.29 (247.28-519.23) | 12.02 (8.2-17.01) | 0.45 (0.34-0.56) |
| Tokelau | 0.06 (0.04-0.09) | 4.48 (2.9-6.61) | 0.04 (0.03-0.06) | 3.33 (2.17-4.88) | -0.95 (-0.99--0.91) |
| Tonga | 2.08 (1.32-3.08) | 4.05 (2.64-6) | 2.35 (1.51-3.42) | 3.08 (1.98-4.53) | -0.97 (-1.04--0.9) |
| Trinidad and Tobago | 13.03 (8.49-19.21) | 1.66 (1.08-2.43) | 24.93 (16.21-36.26) | 1.36 (0.89-1.97) | -0.77 (-0.84--0.71) |
| Tunisia | 818.25 (521.05-1206.98) | 17.45 (11.26-25.66) | 1495.5 (984.99-2180.43) | 12.35 (8.12-17.75) | -1.27 (-1.33--1.2) |
| Turkey | 3444.22 (2279.57-4965.63) | 10.67 (7.07-15.34) | 7035.95 (4670.65-10149.7) | 8.28 (5.49-11.97) | -0.95 (-1.07--0.82) |
| Turkmenistan | 65.25 (44.46-92.87) | 3.81 (2.59-5.37) | 116.4 (78.25-164.33) | 3.41 (2.3-4.81) | -0.44 (-0.56--0.33) |
| Tuvalu | 0.3 (0.19-0.45) | 4.73 (3.04-6.96) | 0.35 (0.22-0.53) | 3.64 (2.35-5.4) | -0.81 (-0.86--0.76) |
| Uganda | 410.17 (273.75-577.78) | 7.3 (4.94-10.2) | 833.66 (552.02-1196.58) | 7.19 (4.73-10.31) | 0.23 (0.02-0.43) |
| Ukraine | 1581.3 (1037.07-2314.57) | 2.26 (1.51-3.26) | 1630.55 (1064.9-2373.96) | 2.06 (1.35-2.99) | -0.49 (-0.53--0.44) |
| United Arab Emirates | 40.77 (26.06-59.13) | 16.27 (10.65-23.28) | 266.45 (171.49-384.87) | 12.79 (8.48-18.04) | -0.76 (-0.81--0.72) |
| United Kingdom | 8801.23 (6120.67-12246.97) | 9.35 (6.49-12.98) | 11937.34 (8294.54-16511.15) | 8.61 (5.96-11.91) | -0.31 (-0.32--0.3) |
| United Republic of Tanzania | 1915.69 (1316.88-2672.82) | 19.94 (13.84-27.76) | 3284.03 (2208.41-4703.98) | 15.49 (10.45-22.36) | -1 (-1.32--0.68) |
| United States of America | 9921.05 (6810.58-14195.46) | 2.93 (2.01-4.18) | 16273.21 (11282.56-23011.23) | 2.65 (1.82-3.75) | -0.47 (-0.63--0.3) |
| United States Virgin Islands | 1.21 (0.78-1.79) | 1.63 (1.07-2.41) | 2.61 (1.64-3.79) | 1.38 (0.89-1.97) | -0.58 (-0.64--0.52) |
| Uruguay | 142.17 (92.98-207.2) | 3.62 (2.39-5.22) | 191.58 (128.57-275.79) | 3.15 (2.11-4.53) | -0.41 (-0.45--0.38) |
| Uzbekistan | 524.21 (347.59-750.95) | 5.05 (3.37-7.22) | 750.31 (495.24-1060.89) | 4.69 (3.13-6.5) | -0.28 (-0.4--0.17) |
| Vanuatu | 2.65 (1.78-3.78) | 4.55 (3.1-6.47) | 5.15 (3.31-7.66) | 3.29 (2.15-4.83) | -1.16 (-1.38--0.95) |
| Venezuela (Bolivarian Republic of) | 333.69 (219.25-482.4) | 3.93 (2.6-5.71) | 936.51 (625.53-1354.38) | 3.4 (2.27-4.94) | -0.39 (-0.48--0.29) |
| Viet Nam | 4044.56 (2757.13-5865.51) | 10.79 (7.39-15.41) | 6508.49 (4417.39-9102.89) | 7.76 (5.31-10.85) | -1.71 (-1.92--1.5) |
| Yemen | 600.47 (385.38-886.43) | 14.42 (9.35-20.87) | 1839.8 (1241.96-2615.77) | 16.34 (11.11-22.92) | 0.32 (0.12-0.52) |
| Zambia | 171.25 (111-246.66) | 7.17 (4.78-10.35) | 252.39 (166.45-372.45) | 4.61 (3.08-6.75) | -1.58 (-1.77--1.39) |
| Zimbabwe | 98.13 (64.02-146.14) | 2.86 (1.87-4.18) | 152.41 (100.47-227.59) | 2.67 (1.79-3.94) | -0.23 (-0.33--0.12) |

AMD, age-related macular degeneration; UI, uncertainty interval; CI, confidence interval; ASR, age-standardized rate; EAPC, estimated annual percentage change; YLDs, years lived with disability.


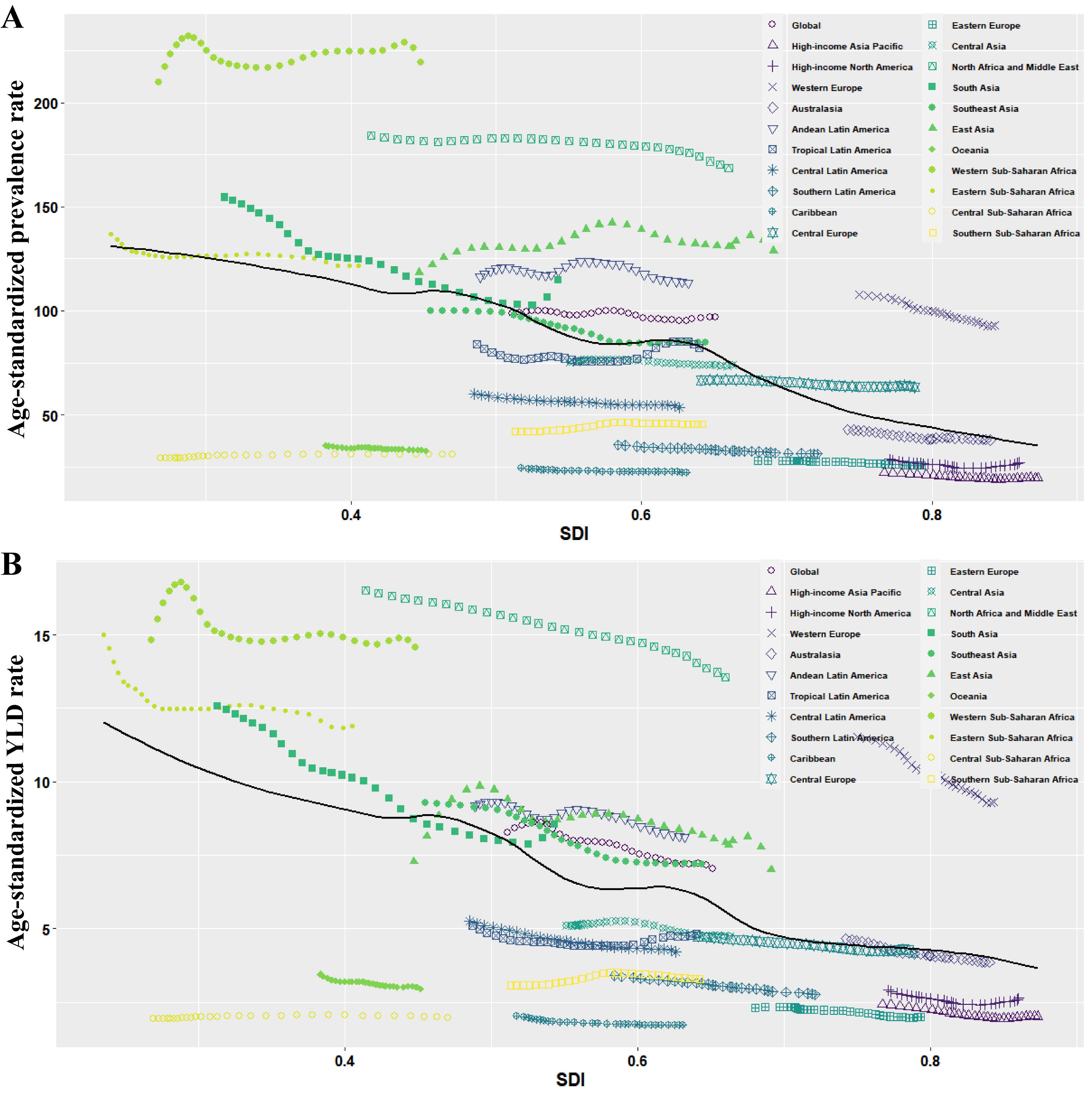


**Figure S1. Global burden of the prevalence and YLDs of AMD by SDI.** (A) The black line represents the average expected relationship between SDIs and age-standardized prevalence rates for AMD based on values from 21 GBD regions from 1990 to 2019; (B) The black line represents the average expected relationship between SDIs and age-standardized YLD rates for AMD based on values from 21 GBD regions from 1990 to 2019. AMD, age-related macular degeneration; SDI, socio-demographic index; GBD, global burden of disease; YLDs, years lived with disability.
